# Cardiovascular disease risk in patients with psoriasis receiving biologics targeting TNF-α, IL-12/23, IL-17, and IL-23: A population-based retrospective cohort study

**DOI:** 10.1371/journal.pmed.1004591

**Published:** 2025-04-17

**Authors:** Teng-Li Lin, Yi-Hsuan Fan, Kuo-Sheng Fan, Chao-Kuei Juan, Yi-Ju Chen, Chun-Ying Wu

**Affiliations:** 1 Department of Dermatology, Dalin Tzu Chi Hospital, Buddhist Tzu Chi Medical Foundation, Chiayi, Taiwan; 2 Ph.D. Program of Interdisciplinary Medicine, National Yang Ming Chiao Tung University, Taipei, Taiwan; 3 Department of Pediatrics, Chung Shan Medical University Hospital, Taichung, Taiwan; 4 Department of Internal Medicine, Division of Chest Medicine, Dalin Tzu Chi Hospital, Buddhist Tzu Chi Medical Foundation, Chiayi, Taiwan; 5 Department of Dermatology, Taichung Veterans General Hospital, Taichung, Taiwan; 6 Department of Post-Baccalaureate Medicine, College of Medicine, National Chung Hsing University, Taichung, Taiwan; 7 Faculty of Medicine and Institute of Clinical Medicine, National Yang Ming Chiao Tung University, Taipei, Taiwan; 8 Institute of Biomedical Informatics, National Yang Ming Chiao Tung University, Taipei, Taiwan; 9 Division of Translational Research, Taipei Veterans General Hospital, Taipei, Taiwan; 10 College of Public Health, China Medical University, Taichung, Taiwan; Leiden University Medical Centre, NETHERLANDS, KINGDOM OF THE

## Abstract

**Background:**

Psoriasis is associated with various cardiovascular diseases (CVDs). The aim of this study was to compare the risk of CVD in patients with psoriasis who were prescribed biologics or oral therapies, and to assess the association between different classes of biologics and CVD risk.

**Methods and Findings:**

This retrospective cohort study utilized the TriNetX Global Collaborative Network (2014–2025). Patients with psoriasis newly prescribed biologics (BIO-cohort) and those newly initiating oral anti-psoriatic drugs without biologic exposure (Non-BIO-cohort) were enrolled. A propensity score-matched analysis was conducted, accounting for age, sex, race, comorbidities, body mass index, serum lipid profile, and inflammatory marker levels. Cardiovascular risk was compared between the BIO- and Non-BIO-cohorts using Cox regression to calculate hazard ratios (HRs) with 95% confidence intervals (CIs). After matching, each cohort comprised 12,732 patients, with approximately 50% being female, a mean age of 57 years, and 55% identifying as White. The 5-year cumulative incidence of any CVDs was significantly lower in the BIO-cohort (10.68%; 95% CI [10.03%, 11.36%]) than in the Non-BIO-cohort (16.17%; 95% CI: [15.34%, 17.05%]) (*p* < 0.001). The BIO-cohort had attenuated risks of any CVDs (HR 0.621; 95% CI [0.571, 0.676]), cerebrovascular diseases (HR 0.616; 95% CI [0.519, 0.731]), arrhythmias (HR 0.632; 95% CI [0.565, 0.706]), inflammatory heart diseases (HR 0.566; 95% CI [0.360, 0.891]), ischemic heart diseases (HR 0.579; 95% CI [0.465, 0.721]), heart failure (HR 0.637; 95% CI [0.521, 0.780]), non-ischemic cardiomyopathy (HR 0.654; 95% CI [0.466, 0.918]), thrombotic disorders (HR 0.570; 95% CI [0.444, 0.733]), peripheral arterial occlusive diseases (HR 0.501; 95% CI [0.383, 0.656]), and major adverse cardiac events (HR 0.697; 95% CI [0.614, 0.792]). Receiving only anti-tumor necrosis factor (TNF)-α (HR 0.886; 95% CI [0.807, 0.973]), anti-interleukin (IL)-17 (HR 0.724; 95% CI [0.599, 0.875]), or anti-IL-23 (HR 0.739; 95% CI [0.598, 0.914]) was associated with reduced risks of any CVDs, whereas no significant association was observed for only anti-IL-12/23 (HR 0.915; 95% CI [0.742, 1.128]). This risk reduction remained consistent across various subgroups, including age (≤45 or >45 years), sex (male or female), regions of research data (the United States, Europe, Middle East and Africa, and Asia-Pacific), and comorbidities (psoriatic arthritis, hypertension, diabetes, hyperlipidemia, overweight or obesity). Eight sensitivity analyses, such as extending the washout period or tightening medication definitions, validated our findings. The main limitation of our study is the observational design, which can only establish associations, not causation.

**Conclusions:**

Patients with psoriasis prescribed biologics exhibited a lower risk of CVDs versus those on oral therapy. Anti-TNF-α, anti-IL-17, and anti-IL-23 were associated with decreased cardiovascular hazards, while anti-IL-12/23 was not.

## Author summary

**Why was this study done?:**

- People with psoriasis have a higher risk of heart disease, possibly due to inflammation caused by certain proteins in the body, such as tumor necrosis factor (TNF)-α, interleukin (IL)-23, and IL-17.
- Biologics used to treat psoriasis, which inhibit TNF-α or the IL-23/IL-17 pathway, have been shown to improve heart health.

**What did the researchers do and find?:**

- We analyzed electronic health records from the TriNetX Network, which includes data from 156 million patients across 142 healthcare organizations in 18 countries.
- We included 12,732 patients with psoriasis on biologics and 12,732 on oral psoriasis drugs, and found that those on biologics had a lower risk of heart diseases over a five-year follow-up period.
- This lower risk was seen across different heart conditions, including stroke, arrhythmias, inflammatory heart diseases, ischemic heart diseases, heart failure, non-ischemic cardiomyopathy, thrombotic disorders, peripheral arterial occlusive diseases, and major adverse cardiac events.
- Patients receiving anti-TNF-α, anti-IL-17, or anti-IL-23 alone had a lower risk of heart disease, while no association was observed for those receiving anti-IL-12/23 alone.

**What do these findings mean?:**

- Biologic treatment may help protect patients with psoriasis from heart diseases.
- More research is needed to understand how biologics targeting TNF-α, IL-17, or IL-23 might reduce heart disease risk and whether this benefit applies to a broader population.
- While this study shows an association between biologics commonly in psoriasis treatment and lower heart disease risk, it does not establish causality.

## Abstract 3

Using electronic health records from the TriNetX network, Teng-Li Lin and colleagues compare the risk of cardiovascular disease in patients with psoriasis who were prescribed biologic versus oral therapies.

## Introduction

Psoriasis (PsO) is a chronic dermatosis characterized by systemic inflammation, which can drive dysfunction in organs beyond the skin. Cardiovascular diseases (CVDs) frequently accompany PsO as comorbidities. Research indicates elevated risks of various CVDs in PsO, including stroke, myocardial infarction (MI), heart failure, atrial fibrillation, and vascular disorders [1–4]. In the European guideline for CVD prevention, PsO has been recognized as a contributing risk factor, amplifying cardiovascular hazard by 1.5 times [5].

The shared immunoinflammatory mechanism underlies the association between PsO and CVDs. The upstream pathogenic cytokine of PsO, tumor necrosis factor-α (TNF-α), can trigger oxidative stress and augment angiotensin effects, resulting in endothelial damage and vasomotor disruption [6,7]. Aberrantly activated interleukin (IL)-23/IL-17 axis, the key factor in PsO [8], is also implicated in atherosclerosis and atheroma instability, thereby causing vessel stiffness and thrombosis [9–11]. The particularly high cardiovascular risk in severe PsO echoes the contribution of psoriatic inflammation to CVD development [1,12].

Evidence suggests that controlling inflammation in PsO with conventional systemic anti-psoriatic drugs could alleviate CVD risk [13–16]. By targeting key psoriatic effectors, biologics offer enhanced efficacy against PsO and potentially heightened benefits in reducing subsequent CVDs. Inhibiting TNF-α or the IL-23/IL-17 pathway has demonstrated improvements in various cardiovascular health indicators [17–23]. Consistent with this, biologic treatment for PsO is linked to a greater mitigation in CVD risk in certain investigations [14,24,25]. Nevertheless, conflicting results also exist across the literature [26–28]. Moreover, the role of newly developed biologics targeting IL-17 or IL-23 on CVDs remains understudied. Another gap is the limited exploration of CVD risk beyond stroke and MI in patients with PsO receiving biologics [14,24–28].

This study aimed to assess the association between biologics and CVD risk in patients with PsO compared to oral anti-psoriatic drugs. We hypothesized that biologic treatments would be associated with a lower risk of CVDs in patients with PsO compared to those on oral therapy.

## Methods

### Data source

This retrospective cohort study utilized data from the Global Collaborative Network within the TriNetX database, spanning 2014–2025. TriNetX is a research platform gathering anonymized, real-time data from multiple healthcare organizations worldwide, with records updated approximately every two weeks. The Global Collaborative Network, a subset of TriNetX, comprises around 156 million patients from 142 healthcare organizations across 18 countries (S1 Text). Available data includes demographics, diagnoses, measurements, medications, and procedures, which are extensively utilized in epidemiologic research [29,30]. Details about the standardized codes used in this study are available in the online S1 Table. Data analysis was concluded on February 6, 2025. Ethical approval was granted by Taichung Veterans General Hospital, Taiwan (No. CE23353C), with informed consent waived due to data de-identification.

### Study design and population

Patients aged 18 years or older with two separate PsO diagnoses, spaced at least 30 days apart, were eligible. This approach, validated in electronic health record databases, helps reduce misclassification and has been used in other high-quality studies [30,31]. The biologic cohort (BIO-cohort) comprised patients prescribed biologics, including anti-TNF-α (infliximab, adalimumab, etanercept, golimumab, and certolizumab), anti-IL-12/23 (ustekinumab), anti-IL-17 (secukinumab, ixekizumab, and brodalimumab), or anti-IL-23 (guselkumab, risankizumab, and tildrakizumab), for a minimum of three times following their PsO diagnosis. Since biologics are typically later-line treatments compared to oral anti-psoriatic drugs, we did not exclude BIO-cohort patients who had previously used oral therapies. The non-biologic cohort (Non-BIO-cohort) encompassed patients prescribed oral anti-psoriatic drugs, including methotrexate, cyclosporin, acitretin, and apremilast, for at least 3 times post-PsO diagnosis, but never biologic use. The 3-treatment minimum aimed to reduce the inclusion of patients with poor adherence. The index date was defined as the initial prescription date of either biologics for the BIO-cohort or oral anti-psoriatic drugs for the Non-BIO-cohort.

We excluded patients with non-psoriatic conditions indicating anti-TNF-α, anti-IL-12/23, anti-IL-17, or anti-IL-23 treatments, such as rheumatoid arthritis, ankylosing spondylitis, juvenile idiopathic arthritis, inflammatory bowel diseases, hidradenitis suppurativa, and uveitis. Patients with CVDs (designated as outcomes of interest) before the index date were excluded. After exclusions, both cohorts underwent 1:1 propensity score matching (PSM) based on current age, age at index, sex, race, comorbidities, and laboratory measurements.

This study is reported as per the Strengthening the Reporting of Observational Studies in Epidemiology (STROBE) guideline (S1 Checklist).

### Covariates

Demographic data, including current age, age at the index date, and sex, were collected. Due to the existence of racial differences in CVD epidemiology, potentially driven by genetic, clinical, and social factors [32], race (but not ethnicity) was accounted to reduce confounding. Race data were obtained through patient self-report or healthcare provider observation and subsequently submitted to the TriNetX Network by healthcare organizations. Comorbidities were analyzed over the 3 years preceding the index date, including hypertension, chronic obstructive pulmonary disease, liver diseases, chronic kidney disease, type 2 diabetes mellitus, hyperlipidemia, depression, sleep disorders, and overweight or obesity. Socioeconomic health hazards and smoking habits were also identified. Measurements were sourced from the TriNetX database, which provides the most recent data before the index date. Cutoff values for various measurement results were set, and TriNetX’s statistical analysis treats these values as categorical covariates. Body mass index (≥27 kg/m²) and laboratory measurements, including triglycerides (≥500 mg/dL), low-density lipoprotein cholesterol (≥190 mg/dL), high-density lipoprotein cholesterol (<40 mg/dL), C-reactive protein (>3 mg/L), and erythrocyte sedimentation rate (>25 mm/h) in blood, were assessed. Both current age and age at index were included as continuous variables in the matching process to mitigate period effects. Other categorical covariates with >3% prevalence in either cohort were incorporated into the PSM to retain as many participants as possible while ensuring proper matching and avoiding overfitting.

### Outcomes

The primary outcome was the first incident of any CVDs within the TriNetX network, defined as the composite of various categories of cardiovascular complications, including: (1) cerebrovascular diseases (stroke and transient ischemic attacks), (2) arrhythmias (atrial fibrillation, atrial flutter, tachycardia, bradycardia, and ventricular arrhythmias), (3) inflammatory heart diseases (pericarditis and myocarditis), (4) ischemic heart diseases (acute coronary disease, MI, ischemic cardiomyopathy, and angina), (5) heart failure, (6) non-ischemic cardiomyopathy, (7) thrombotic disorders (pulmonary embolism and superficial or deep vein thrombosis), (8) peripheral arterial occlusive disease, and (9) cardiac arrest or cardiogenic shock. Additionally, we separately analyzed these disease categories and the major adverse cardiac events (MACEs), described as instances of MI, stroke, heart failure, ventricular arrhythmia, and cardiac arrest.

As a secondary outcome, we examined medications commonly used for these CVDs within the database, including thrombolytics, antiplatelets, anticoagulants, antiarrhythmics, antianginals, vasodilators, *β*-blockers, cardiac-selective calcium channel blockers, angiotensin-converting enzyme inhibitors, angiotensin II receptor blockers, diuretics, sympathomimetics, digitalis glycoside, and cilostazol. Cardiovascular procedures, such as cardiac catheterization, coronary artery bypass graft, and pacemaker implantation, were also analyzed.

In clinical practice, a 3–4-month treatment period is generally considered a reasonable timeframe for PsO treatments to show efficacy, as reflected in pivotal trials for biologics, where primary efficacy endpoints are typically assessed at 12–16 weeks [33]. To evaluate the role of treatment on CVDs in patients with PsO, we implemented a 6-month washout period after the index date, during which outcomes were not considered. Participants were followed for up to five years or until experiencing the outcomes of interest, death, or their last database entry.

### Subgroup analyses

To assess the relationship between different biologic mechanisms and CVD risk, we categorized patients prescribed only one class of biologics: either anti-TNF-α, anti-IL-12/23, anti-IL-17, or anti-IL-23. Those prescribed two different classes of biologics were not included. These patients were compared separately to those on oral therapy for the risk of CVDs. Since different biologics, even within the same class, can still result in varying therapeutic responses, we further applied an individual comparator-restricted approach, comparing each biologic separately with oral anti-psoriatic drugs. Patients who had used two or more different biologics were excluded from this analysis.

We also examined the association between biologics and CVDs versus oral therapy across subgroups distinguished by age (≤45 years or >45 years), sex (male or female), and presence of comorbidities such as psoriatic arthritis (PsA), hypertension, type 2 diabetes mellitus, hyperlipidemia, and overweight or obesity. Moreover, we analyzed data from different regions: the United States of America (USA), Europe, the Middle East, Africa (EMEA), and the Asia-Pacific region (S1 Text).

### Sensitivity analyses

To reinforce our primary results, we employed eight sensitivity analyses. Model 1 extended the washout period to 1 year, mitigating the influence of delayed coding of CVDs and potential protopathic bias. Model 2 included death as part of each outcome to address the competing risk of mortality [34]. Model 3 excluded BIO-cohort patients ever prescribed oral anti-psoriatic drugs, ensuring that the primary analysis results genuinely reflect the association between biologics and CVD risk. Model 4 excluded Non-BIO-cohort patients who used cyclosporine, which is associated with dyslipidemia and hypertension side effects, known to increase CVD risk [14,35]. Model 5 excluded patients with apremilast use from the Non-BIO-cohort, limiting this group to conventional anti-psoriatic drugs. Model 6 required at least two prescriptions spaced 6 months apart in both cohorts, in addition to the initial three prescription records, to further improve treatment adherence among included patients. Model 7 additionally included prior use of CVD medications—including thrombolytics, platelet aggregation inhibitors, antiarrhythmics, antianginals, vasodilators, beta-blockers, calcium channel blockers, diuretics, sympathomimetics, digitalis glycosides, and cilostazol—as a matching criterion to reduce confounding. In Model 8, patients with prior CVDs were retained, incorporating these diseases as an additional matching criterion. This approach minimizes bias resulting from the selection of inherently less susceptible patients in either cohort [36].

### Statistical analysis

We employed PSM on the TriNetX platform to adjust for cohort differences without replacement (S2 Text). The propensity scores were computed using logistic regression in scikit-learn, yielding values between 0 and 1. To match participants, greedy nearest neighbor matching was applied with a caliper of 0.1 of the pooled standard deviation, and cohorts were considered adequately matched when the standardized differences were less than 0.1. Outcome incidence was evaluated using the Kaplan–Meier estimator and differences assessed via the log-rank test. Hazard ratios (HRs) for outcomes were determined using the Cox regression. Missing data were handled in accordance with TriNetX platform protocols, which include specific imputations for encounter identifiers, encounter start and end dates, estimated glomerular filtration rate, and oncology diagnoses, with all other missing values remaining unaltered (S3 Text). Computational tasks were performed in R (version 4.3.1), except for PSM and the log-rank test, which were conducted in TriNetX. Statistical significance was set at a two-sided *p*-value <  0.05, with 95% confidence intervals (CIs) excluding 1.

## Results

### Demographic characteristics

**Fig 1:**
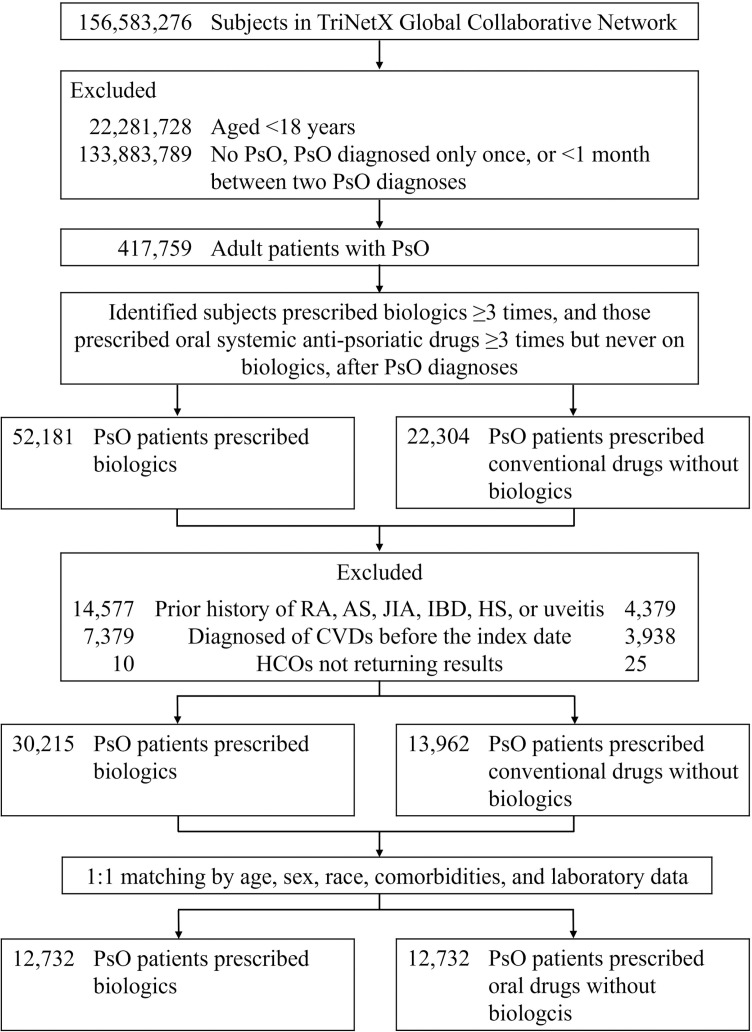
Flow chart of the patient selection. Abbreviation: PsO, psoriasis; RA, rheumatoid arthritis; AS, ankylosing spondylitis; JIA, juvenile idiopathic arthritis; IBD, inflammatory bowel diseases; HS, hidradenitis suppurativa; CVDs, cardiovascular diseases; HCOs, healthcare organizations.

Fig 1 illustrates our cohort selection process. Within the TriNetX Global Collaborative Network, we identified 52,181 patients with PsO-prescribed biologics and 22,304 receiving oral anti-psoriatic drugs without biologic exposure. After exclusions and PSM, both the BIO- and the Non-BIO-cohort comprised 12,732 patients. The demographic data before and after matching are presented in Tables 1 and S2, respectively.

**Table 1:** Demographic characteristics of study subjects after PSM ^*^ .

	BIO-cohort (*N* = 12,732)	Non-BIO-cohort (*N* = 12,732)	Std. diff.^†^
Age, years, mean ± SD			
Current age	56.7 ± 15.2	57.3 ± 16.4	0.041
Age at index	50.2 ± 14.8	50.8 ± 15.9	0.040
Female, *N* (%)	6,411 (50.4%)	6,243 (49.0%)	0.026
Race, *N* (%)^‡^			
White	6,831 (53.7%)	7,003 (55.0%)	0.027
Black or African American	451 (3.5%)	451 (3.5%)	<.001
Asian	1,481 (11.6%)	1,462 (11.5%)	0.005
Unavailable	3,003 (23.6%)	3,406 (26.8%)	0.073
Follow-up, years, mean ± SD	4.8 ± 0.9	4.6 ± 1.1	0.160
Comorbidity, *N* (%)			
Essential hypertension	2,086 (16.4%)	2,236 (17.6%)	0.031
Chronic obstructive pulmonary disease	242 (1.9%)	237 (1.9%)	0.003
Liver diseases	462 (3.6%)	484 (3.8%)	0.009
Chronic kidney disease	209 (1.6%)	211 (1.7%)	0.001
Type 2 diabetes mellitus	911 (7.2%)	957 (7.5%)	0.014
Hyperlipidemia	1,325 (10.4%)	1,383 (10.9%)	0.015
Depression	825 (6.5%)	834 (6.6%)	0.003
Sleep disorders	896 (7.0%)	949 (7.5%)	0.016
Overweight or obesity	1,137 (8.9%)	1,139 (8.9%)	0.001
Smoking, *N* (%)^§^			
Tobacco use	146 (1.1%)	147 (1.2%)	0.001
Nicotine dependence	592 (4.6%)	622 (4.9%)	0.011
Socioeconomic challenges, *N* (%)^§^	112 (0.9%)	103 (0.8%)	0.008
Laboratory measurements¶			
Body mass index (kg/m^2^), mean ± SD	30.5 ± 7.5	30.6 ± 7.4	0.011
≥27 kg/m^2^, *N* (%)	3,469 (27.2%)	3,483 (27.4%)	0.002
TG in blood (mg/dL), mean ± SD	139.3 ± 89.2	141.0 ± 100.0	0.018
≥500 mg/dL, *N* (%)	60 (0.5%)	58 (0.5%)	0.002
LDL-C in blood (mg/dL), mean ± SD	108.5 ± 35.6	108.9 ± 35.5	0.011
≥190 mg/dL, *N* (%)	96 (0.8%)	102 (0.8%)	0.005
HDL-C in blood (mg/dL), mean ± SD	51.6 ± 19.5	50.9 ± 20.0	0.034
<40 mg/dL	891 (7.0%)	959 (7.5%)	0.021
CRP in blood (mg/L), mean ± SD	9.0 ± 22.2	10.6 ± 24.1	0.068
>3 mg/L, *N* (%)	1,216 (9.6%)	1,287 (10.1%)	0.019
ESR in blood (mm/h), mean ± SD	17.1 ± 18.1	19.2 ± 19.2	0.112
>25 mm/h, *N* (%)	566 (4.4%)	582 (4.6%)	0.006

* PSM was conducted based on current age, age at index, sex, race, and covariates that had a prevalence of over 3% in any cohort (including hypertension, liver diseases, type 2 diabetes mellitus, hyperlipidemia, depression, sleep disorders, overweight or obesity, nicotine dependence, and subjects with BMI ≥ 27 kg/m^2^, HDL-C < 40 mg/dL, CRP > 3 mg/L, and ESR > 25 mm/h).

† Cohorts were considered well-matched when standardized differences were below 0.1.

‡ Races not fully represented include the sparsely numbered American Indian, Alaska Native, Native Hawaiian, and other Pacific Islander.

§ Persons with tobacco use, nicotine dependence, and potential health hazards related to socioeconomic and psychosocial circumstances were identified with ICD-10-CM Z72.0, F17, and Z55-Z65, respectively.

¶ Laboratory test results were based on the most recent measurements prior to the index event.

PSM, propensity score matching; BIO-cohort, biologic cohort; Non-BIO-cohort, non-biologic cohort; Std. diff., standardized difference; SD, standard deviation; *N*, number; TG, triglyceride; LDL-C, low density lipoprotein cholesterol; HDL-C, high density lipoprotein cholesterol; CRP, C-reactive protein; ESR, erythrocyte sedimentation rate.

For the BIO-cohort, the mean ages were 56.7 years (standard deviation [SD] 15.2) currently and 50.2 years (SD 14.8) at the index; the Non-BIO-cohort had respective averages of 57.3 (SD 16.4) and 50.8 (SD 15.9) years. The mean follow-up duration was 4.8 years (SD 0.9) for the BIO-cohort and 4.6 years (SD 1.1) for the Non-BIO-cohort. Both cohorts exhibited similar sex and racial distributions, with approximately half of the patients being female and 55% being White. Well-matched characteristics in comorbidities, smoking status, socioeconomic challenges, and laboratory results were observed. Hypertension, hyperlipidemia, and overweight or obesity were the most prevalent conditions.

### Incidence and risk of CVDs and associated therapy utilization

**Fig 2:**
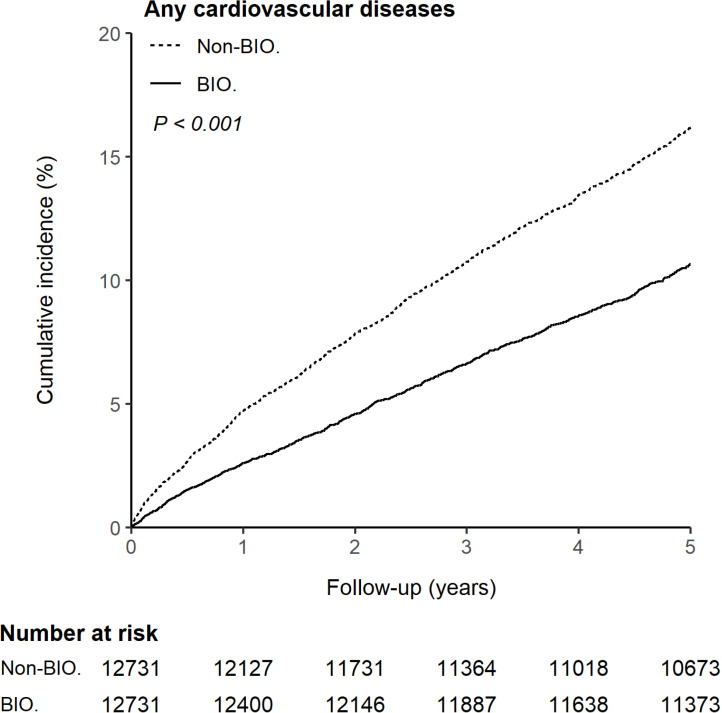
Cumulative incidence of any cardiovascular diseases in the BIO-cohort vs. the Non-BIO-cohort. The differences between the two study cohorts were determined by log-rank test. Abbreviation: BIO., biologic cohort; Non-BIO., non-biologic cohort.

During observation, 950 patients (7.5%) in the BIO-cohort and 1,281 patients (10.1%) in the Non-BIO-cohort developed CVDs (*p* < 0.001) (S3 Table). The BIO-cohort demonstrated a significantly lower 5-year cumulative incidence of any CVDs (10.68%; 95% CI [10.03%, 11.36%]) compared to the Non-BIO-cohort (16.17%; 95% CI [15.34%, 17.05%]) (*p* <  0.001) (Fig 2). There were also lower occurrences of nearly all categories of CVDs in the BIO-cohort, with 12 out of 14 CVD-associated treatments showing fewer events, 6 of which demonstrated a significant reduction in utilization compared to the Non-BIO-cohort (S3 Table and S1 Fig).

**Fig 3:**
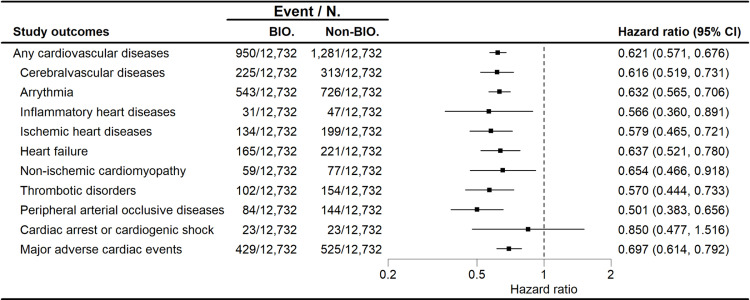
Forest plot depicting hazard ratios for the development of cardiovascular diseases. Among patients with psoriasis, biologic treatment was found to be associated with a decreased risk of any cardiovascular diseases and almost all categories of cardiovascular diseases. Abbreviation: BIO., biologic cohort; Non-BIO., non-biologic cohort; CI, confidence interval.

In Cox regression, the BIO-cohort had significantly attenuated risks of any CVDs (HR 0.621; 95% CI [0.571, 0.676]), cerebrovascular diseases (HR 0.616; 95% CI [0.519, 0.731]), arrythmias (HR 0.632; 95% CI [0.565, 0.706]), inflammatory heart diseases (HR 0.566; 95% CI [0.360, 0.891]), ischemic heart diseases (HR 0.579; 95% CI [0.465, 0.721]), heart failure (HR 0.637; 95% CI [0.521, 0.780]), non-ischemic cardiomyopathy (HR 0.654; 95% CI [0.466, 0.918]), thrombotic disorders (HR 0.570; 95% CI [0.444, 0.733]), peripheral arterial occlusive diseases (HR 0.501; 95% CI [0.383, 0.656]), and MACEs (HR 0.697; 95% CI [0.14, 0.792]) (Fig 3). The risk of cardiac arrest or cardiogenic shock (HR 0.850; 95% CI [0.477, 1.516]) was lower with biologics than oral therapy, though the 95% CIs included 1. Furthermore, reduced risks in utilizing CVD-associated treatments were also observed in the BIO-cohort versus the Non-BIO-cohort, with significant differences found in antiplatelets (HR 0.769; 95% CI [0.698, 0.848]), anticoagulants (HR 0.848; 95% CI [0.794, 0.906]), antiarrhythmics (HR 0.661; 95% CI [0.485, 0.900]), antianginals (HR 0.663; 95% CI [0.549, 0.801]), vasodilators for cardiac diseases (HR 0.659; 95% CI [0.546, 0.796]), β-blockers (HR 0.795; 95% CI [0.727, 0.869]), cardiac-selective calcium channel blockers (HR 0.545; 95% CI [0.418, 0.711]), diuretics (HR 0.865; 95% CI [0.801, 0.935]), and cilostazol (HR 0.240; 95% CI [0.089, 0.646]) (S2 Fig).

### Subgroup analyses

**Fig 4:**
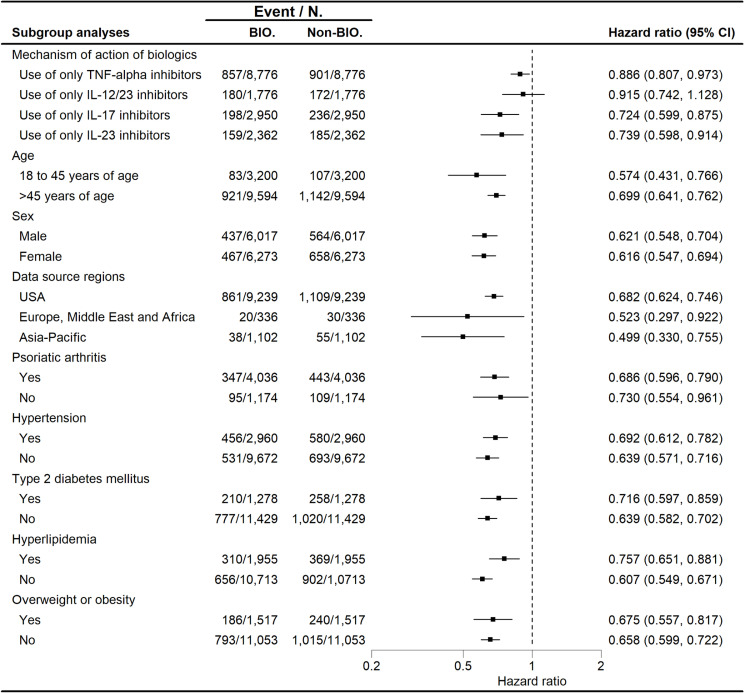
Forest plot depicting hazard ratios for the development of any cardiovascular diseases stratified by biologic classes, demographics, regions of data source, and comorbidities. Patients with psoriasis prescribed only anti-TNF-α, anti-IL-17, or anti-IL-23 showed lower cardiovascular disease risks, while no significant reduction was seen with anti-IL-12/23 alone. This risk reduction remained significant across diverse demographics and comorbidities with biologic prescriptions. Abbreviation: BIO., biologic cohort; Non-BIO., non-biologic cohort; CI, confidence interval.

The cumulative incidence of any CVDs over 5 years for patients prescribed different classes of biologics versus oral anti-psoriatic drugs is presented in S3 Fig. Patients receiving only anti-TNF-α (HR 0.886; 95% CI [0.807, 0.973]), anti-IL-17 (HR 0.724; 95% CI [0.599, 0.875]), or anti-IL-23 (HR 0.739; 95% CI [0.598, 0.914]) all showed significantly lower risks of any CVDs. However, no significant association was observed for only anti-IL-12/23 (HR 0.915; 95% CI [0.742, 1.128]) (Fig 4). Risk was generally lower for specific category of CVDs within these subgroups of single-class biologics, though the case numbers were limited, with statistical significance observed in just a small subset of events (S4 Table). When comparing individual biologics separately with oral anti-psoriatic drugs, the sample sizes were further reduced. Only a few outcomes showed significant differences, accompanied by wide CIs (S5 Table).

Subgroup analyses across demographic and clinical categories uniformly demonstrated the association of biologic therapy with reduced CVD risk, even among individuals older than 45 years (HR 0.699; 95% CI [0.641, 0.762]), males (HR 0.621; 95% CI [0.548, 0.704]), patients with coexisting PsA (HR 0.686; 95% CI [0.596, 0.790]), or those with comorbid hypertension (HR 0.692; 95% CI [0.612, 0.782]), type 2 diabetes mellitus (HR 0.716; 95% CI [0.597, 0.859]), hyperlipidemia (HR 0.757; 95% CI [0.651, 0.881]), or overweight or obesity (HR 0.675; 95% CI [0.557, 0.817]) (Fig 4). This reduction in risk was also consistent across patient data from the USA (HR 0.682; 95% CI [0.624, 0.746]), EMEA (HR 0.523; 95% CI [0.297, 0.922]), and the Asia-Pacific region (HR 0.499; 95% CI [0.330, 0.755]).

### Sensitivity analyses

All sensitivity analyses corroborated the primary findings (S4 Fig). From Models 1 to 7, each respectively representing extending the washout period (HR 0.628; 95% CI [0.575, 0.686]), accounting for competing mortality (HR 0.600; 95% CI [0.554, 0.650]), excluding BIO-cohort patients previously prescribed oral therapies (HR 0.654; 95% CI [0.595, 0.718]), excluding cyclosporine users from the Non-BIO-cohort (HR 0.667; 95% CI [0.611, 0.729]), restricting the Non-BIO-cohort to conventional drugs (HR 0.627; 95% CI [0.568, 0.692]), refining prescription criteria to include patients with better adherence (HR 0.621; 95% CI [0.571, 0.676]), and additionally matching for prior CVD medication use (HR 0.651; 95% CI [0.600, 0.708]), biologics were associated with a decreased risk of any CVDs. Notably, in Model 8, by including patients with prior CVDs as an additional matching criterion, the significant risk reduction (HR 0.830; 95% CI [0.790, 0.871]) should be interpreted as alleviating CVD exacerbations or newly occurring events necessitating medical visits.

## Discussions

In this cohort study using a global health research network, patients with PsO-prescribed biologics exhibited a reduced risk of incident CVDs compared to those receiving oral anti-psoriatic drugs. Anti-TNF-α, anti-IL-17, and anti-IL-23 were associated with decreased cardiovascular hazards, while the anti-IL-12/23 was not. The biologic benefits against CVDs were consistent across patients with PsO of different age groups, sexes, regions, and comorbidities.

Meta-analyses of randomized controlled trials have demonstrated no difference or even an increased risk of CVDs in patients with PsO receiving biologic treatment [26–28]. However, the result may not reflect real-world scenarios due to short controlled periods in trials and the inclusion of non-licensed biologics in the analyses. In contrast, cohort studies suggested a neutral or protective association between biologics and CVDs in PsO [14,24,25], but limitations included small sample sizes and lack of confounders such as body fat and laboratory measurements. In this study, we compared 12,732 patients with PsO-prescribed biologics to those receiving oral therapy. Comorbidities, smoking status, body mass index, lipid profiles, and inflammation biomarkers, all related to CVD development [37–39], were well-matched. Patients on biologics had a 38% reduced risk of CVDs over the 5-year follow-up. The results support the link between psoriatic inflammation and CVDs, highlighting the potential of targeted anti-inflammatory therapy for cardiovascular protection.

Despite the known association of PsO with various CVDs [1–4], previous literature mainly focused on the relationship between biologic use and stroke or MI [14,24–28]. Our study further demonstrated the attenuated risk of multiple CVD categories with biologics. Research has indicated alleviated atherosclerosis and coronary inflammation in biologic-treated patients [17,20,40,41], aligning with our findings of decreased risk of cerebrovascular diseases, ischemic heart diseases, and peripheral arterial occlusive disease in the BIO-cohort. Diminished oxidative stress and improved endothelial function after initiating biologics in PsO mouse models and humans may explain the reduced risk of arrhythmias and thrombotic disorders in our study [18,21]. Additionally, biologics have been shown to enhance myocardial function in echocardiography [22, 23], reinforcing their link to the mitigated risk of inflammatory heart diseases, heart failure, and cardiomyopathy. The decreased use of various CVD-associated therapies in the BIO-cohort may also reflect a potential relationship between biologics and a lower risk across multiple CVD categories.

This study investigates the association between novel biologics targeting IL-17 and IL-23 and CVD risk in PsO, alongside earlier agents targeting TNF-α and IL-12/23, in comparison to oral therapies. Patients solely prescribed anti-TNF-α had an 11% lower CVD risk, contrasting with previous findings of a 40%–50% reduction [14,24,25]. The numerical discrepancy may result from past research defining non-systemic therapy as the reference group or focusing only on MI as the outcome. Nonetheless, anti-TNF-α consistently demonstrated significant advantages in reducing CVDs. While IL-17 confers pro-atherosclerotic functions [10,11], experimental investigations also observed its anti-atherosclerotic effects, both in animal models and humans [42,43]. However, in patients with PsO, IL-17 inhibition is generally associated with improvements in cardiovascular soft endpoints regarding endothelial health, atheroma burden, and myocardial function [21,44,45]. Our result also suggests a lower risk of CVDs in PsO among patients receiving anti-IL-17. Information about CVD risk in the context of IL-23 inhibition remains scarce. Although IL-23 promotes oxidative stress and atherosclerosis [9,10], limited animal studies have not shown benefits in ameliorating atheroma from IL-23 blockage [46,47]. Our findings show a link between anti-IL-23 and a lower CVD risk in PsO clinically, but its precise mechanism requires clarification.

In this study, patients prescribed anti-IL-12/23 did not exhibit a significant reduction in CVD risk compared to those on oral therapy, consistent with prior research [14,26]. Given the postulated association between psoriatic inflammation and CVD development, the decreased CVD risk with anti-IL-23, but not anti-IL-12/23, may stem from the superior efficacy of anti-IL-23 in treating PsO compared to anti-IL-12/23 [33,48]. Another possibility could be the destabilization of atheroma during the initial phase of anti-IL-12/23 therapy [49]. However, this temporary pro-atherogenic response diminishes with continued treatment [50]. Clinical evidence also shows improvements in arterial and myocardial function in patients with PsO receiving ustekinumab [40,51]. Overall, anti-IL-12/23 is still considered potentially cardioprotective for patients with PsO [35], and our results suggest its protection is comparable to oral anti-psoriatic drugs.

Different biologics, even within the same class, vary in origin, structure, and pharmacokinetics, resulting in differences in immunogenicity, efficacy, and adverse effects, which may be associated with varying cardiovascular risks among their users. [52]. For instance, while ustekinumab has shown no link to MACE risk in literature or our study, another anti-IL-12/23 [14,26], briakinumab, was discontinued in 2011 due to MACE concerns in early trials [53]. Current evidence supports the cardiovascular safety of anti-IL-23 [53]. However, a recent FDA Adverse Event Reporting System analysis suggested a potential cerebrovascular risk with risankizumab, possibly due to impaired anti-atherosclerotic activity from reduced IL-17 levels following anti-IL-23 [54,55]. In our study, a comparator-restricted analysis showed a significant reduction in cerebrovascular diseases among risankizumab users versus oral therapy. Nevertheless, the wide CI reflects limited sample size and reduced estimate precision, highlighting the need for larger studies. Beyond drug class and mechanism, subgroup analyses consistently showed reduced CVD risk across demographics, regions, and comorbidities, supporting the generalizability of this association.

Our study has several strengths. Utilizing a large real-world database mitigates selection bias and enhances statistical robustness. The study design adopts a new-user, active comparator approach, considers detailed confounder information, and performs multiple subgroup analyses. Sensitivity tests bolster confidence in the decreased CVD risk associated with biologics: extending the washout period and accounting for competing mortality reinforce the temporal relationship; excluding oral therapy users from the BIO-cohort and cyclosporine or apremilast users from the Non-BIO-cohort increases specificity and provides more clinically insights; tightening medication definitions and considering other CVD drugs supports the validity; incorporating CVDs as matching criteria prevents the depletion of susceptible effect [36].

Our study has several limitations. Firstly, as an observational study, we cannot establish direct causality but only report a lower CVD risk in patients with PsO-prescribed biologic versus oral therapy. Secondly, information such as PsO severity, the basis for treatment selection, and medical records from institutions outside the TriNetX system were unavailable. To address this, we chose patients receiving oral therapy as the reference group to ensure comparable PsO severity between cohorts, aided by the matching to balance their characteristics. The large-scale nature of this big data analysis also helps reduce bias from differences in treatment selection rationale and unrecorded medical interventions outside the database. However, since biologics are often a later line of therapy, the BIO-cohort may have had higher PsO severity than the Non-BIO-cohort. If so, considering the link between PsO severity and CVD risk [1,12], the current study may underestimate the protective benefit of biologics in reducing CVD risk. Thirdly, coding errors could exist in the database, but we sought to limit misclassification by including only patients with two PsO diagnoses separated by at least a month. Moreover, we evaluated not just CVDs but also CVD-associated treatments. Fourthly, multiple comparisons may increase the risk of chance outcomes, and statistical corrections are not applicable due to the constraints of the TriNetX database’s analytical methods. Nevertheless, the observed reduced CVD risk in biologic-treated patients is biologically plausible and consistent with existing evidence, supporting the reliability of our findings. Finally, the TriNetX database cannot capture time-related dynamics such as treatment duration, cumulative dosage, timing of intervention, or treatment response. Despite this limitation to time-fixed medication exposure analyses, this study provides preliminary but valuable evidence linking biologic therapy to reduced CVD risk in PsO. However, the findings should be interpreted cautiously, as the primary results reflect the overall association of any biologics ever used with subsequent cardiovascular risk, and the study does not compare the relative association of different biologic classes with this risk. Further research is needed to evaluate the impact of dosage, duration, and treatment sequencing across biologic therapies.

In conclusion, this observational study revealed a reduced risk of CVDs in patients with PsO-prescribed biologics versus oral therapy. The risk reduction persisted with anti-TNF-α, anti-IL-17, and anti-IL-23 alone, but not with anti-IL-12/23.

## Supporting information

S1 Text Data acquisition. (PDF)

S2 Text Propensity score matching. (PDF)

S3 Text Handling of missing data. (PDF)

S1 Table Codes for all diagnoses, laboratory tests, and medications in our analysis. (PDF)

S2 Table Demographic characteristics of study subjects before propensity score matching (PSM). (PDF)

S3 Table Outcome events among patients in the BIO-cohort and Non-BIO-cohort. (PDF)

S4 Table Risk of specific category of cardiovascular diseases in subgroups prescribed single class biologics versus those on conventional systemic anti-psoriatic drugs. (PDF)

S5 Table Cardiovascular disease risk in patients with psoriasis receiving a single biologic versus oral anti-psoriatic drugs after 1:1 propensity score matching. (PDF)

S1 Fig Cumulative incidence of specific category of cardiovascular diseases in the BIO-cohort vs. the Non-BIO-cohort. (PDF)

S2 Fig Forest plot depicting hazard ratios for the utilization of cardiovascular disease-associated treatments. (PDF)

S3 Fig Cumulative incidence of any cardiovascular diseases in users of different biologic classes vs. the Non-BIO-cohort. (PDF)

S4 Fig Forest plot depicting hazard ratios for the development of any cardiovascular diseases in eight sensitivity analyses. (PDF)

S1 Checklist STROBE Statement. (PDF)

## Abbreviations

**:**
CIs: confidence intervals
CVDs: cardiovascular diseases
EMEA: Europe, the Middle East, Africa
HRs: hazard ratios
IL: interleukin
MACEs: major adverse cardiac events
MI: myocardial infarction
PSM: propensity score matching
PsO: psoriasis
SD: standard deviation
STROBE: Strengthening the Reporting of Observational Studies in Epidemiology
TNF-α: tumor necrosis factor-α

## References

[1] Gelfand JM, Neimann AL, Shin DB, Wang X, Margolis DJ, Troxel AB. Risk of myocardial infarction in patients with psoriasis. JAMA. 2006;296(14):1735–41. doi: 10.1001/jama.296.14.1735 17032986

[2] Khalid U, Ahlehoff O, Gislason GH, Kristensen SL, Skov L, Torp-Pedersen C, et al. Psoriasis and risk of heart failure: a nationwide cohort study. Eur J Heart Fail. 2014;16(7):743–8. doi: 10.1002/ejhf.113 24903077

[3] Ahlehoff O, Gislason GH, Jørgensen CH, Lindhardsen J, Charlot M, Olesen JB, et al. Psoriasis and risk of atrial fibrillation and ischaemic stroke: a Danish Nationwide Cohort Study. Eur Heart J. 2012;33(16):2054–64. doi: 10.1093/eurheartj/ehr285 21840930

[4] Lutsey PL, Prizment AE, Folsom AR. Psoriasis is associated with a greater risk of incident venous thromboembolism: the Iowa Women’s Health Study. J Thromb Haemost. 2012;10(4):708–11. doi: 10.1111/j.1538-7836.2012.04646.x 22284895 PMC3319282

[5] Piepoli MF, Hoes AW, Agewall S, Albus C, Brotons C, Catapano AL, et al. 2016 European Guidelines on cardiovascular disease prevention in clinical practice: The Sixth Joint Task Force of the European Society of Cardiology and Other Societies on Cardiovascular Disease Prevention in Clinical Practice (constituted by representatives of 10 societies and by invited experts) Developed with the special contribution of the European Association for Cardiovascular Prevention & Rehabilitation (EACPR). Eur Heart J. 2016;37(29):2315–81. doi: 10.1093/eurheartj/ehw106 27222591 PMC4986030

[6] Yoshizumi M, Perrella M, Burnett JJ, Lee M. Tumor necrosis factor downregulates an endothelial nitric oxide synthase mRNA by shortening its half-life. Circ Res. 1993;73(1):205–9. 7685252 10.1161/01.res.73.1.205

[7] Sriramula S, Haque M, Majid DSA, Francis J. Involvement of tumor necrosis factor-alpha in angiotensin II-mediated effects on salt appetite, hypertension, and cardiac hypertrophy. Hypertension. 2008;51(5):1345–51. doi: 10.1161/HYPERTENSIONAHA.107.102152 18391105 PMC2736909

[8] Blauvelt A, Chiricozzi A. The immunologic role of IL-17 in psoriasis and psoriatic arthritis pathogenesis. Clin Rev Allergy Immunol. 2018;55(3):379–90. doi: 10.1007/s12016-018-8702-3 30109481 PMC6244934

[9] Subramanian M, Thorp E, Tabas I. Identification of a non-growth factor role for GM-CSF in advanced atherosclerosis: promotion of macrophage apoptosis and plaque necrosis through IL-23 signaling. Circ Res. 2015;116(2):e13-24. doi: 10.1161/CIRCRESAHA.116.304794 25348165 PMC4297527

[10] Abbas A, Gregersen I, Holm S, Daissormont I, Bjerkeli V, Krohg-Sørensen K, et al. Interleukin 23 levels are increased in carotid atherosclerosis: possible role for the interleukin 23/interleukin 17 axis. Stroke. 2015;46(3):793–9. doi: 10.1161/STROKEAHA.114.006516 25649806

[11] Lockshin B, Balagula Y, Merola JF. Interleukin 17, inflammation, and cardiovascular risk in patients with psoriasis. J Am Acad Dermatol. 2018;79(2):345–52. doi: 10.1016/j.jaad.2018.02.040 29477740

[12] Ogdie A, Yu Y, Haynes K, Love TJ, Maliha S, Jiang Y, et al. Risk of major cardiovascular events in patients with psoriatic arthritis, psoriasis and rheumatoid arthritis: a population-based cohort study. Ann Rheum Dis. 2015;74(2):326–32. doi: 10.1136/annrheumdis-2014-205675 25351522 PMC4341911

[13] Prodanovich S, Ma F, Taylor JR, Pezon C, Fasihi T, Kirsner RS. Methotrexate reduces incidence of vascular diseases in veterans with psoriasis or rheumatoid arthritis. J Am Acad Dermatol. 2005;52(2):262–7. doi: 10.1016/j.jaad.2004.06.017 15692471

[14] Ahlehoff O, Skov L, Gislason G, Gniadecki R, Iversen L, Bryld LE, et al. Cardiovascular outcomes and systemic anti-inflammatory drugs in patients with severe psoriasis: 5-year follow-up of a Danish nationwide cohort. J Eur Acad Dermatol Venereol. 2015;29(6):1128–34. doi: 10.1111/jdv.12768 25303139

[15] Boehncke S, Fichtlscherer S, Salgo R, Garbaraviciene J, Beschmann H, Diehl S, et al. Systemic therapy of plaque-type psoriasis ameliorates endothelial cell function: results of a prospective longitudinal pilot trial. Arch Dermatol Res. 2011;303(6):381–8. doi: 10.1007/s00403-010-1108-6 21170539

[16] Mahmoudi MJ, Saboor-Yaraghi A-A, Zabetian-Targhi F, Siassi F, Zarnani AH, Eshraghian MR, et al. Vitamin A decreases cytotoxicity of oxidized low-density lipoprotein in patients with atherosclerosis. Immunol Invest. 2016;45(1):52–62. doi: 10.3109/08820139.2015.1095208 26700065

[17] Elnabawi YA, Oikonomou EK, Dey AK, Mancio J, Rodante JA, Aksentijevich M, et al. Association of biologic therapy with coronary inflammation in patients with psoriasis as assessed by perivascular fat attenuation index. JAMA Cardiol. 2019;4(9):885–91. doi: 10.1001/jamacardio.2019.2589 31365032 PMC6669789

[18] Schüler R, Brand A, Klebow S, Wild J, Veras F, Ullmann E. Antagonization of IL-17A attenuates skin inflammation and vascular dysfunction in mouse models of psoriasis. J Invest Dermatol. 2019;139(3):638–47. 30367871 10.1016/j.jid.2018.09.021

[19] Smith E, Prasad K-MR, Butcher M, Dobrian A, Kolls JK, Ley K, et al. Blockade of interleukin-17A results in reduced atherosclerosis in apolipoprotein E-deficient mice. Circulation. 2010;121(15):1746–55. doi: 10.1161/CIRCULATIONAHA.109.924886 20368519 PMC2929562

[20] Jókai H, Szakonyi J, Kontár O, Marschalkó M, Szalai K, Kárpáti S. Impact of effective tumor necrosis factor-alfa inhibitor treatment on arterial intima-media thickness in psoriasis: results of a pilot study. J Am Acad Dermatol. 2013;69(4):523–9. 23891393 10.1016/j.jaad.2013.06.019

[21] von Stebut E, Reich K, Thaçi D, Koenig W, Pinter A, Körber A. Impact of secukinumab on endothelial dysfunction and other cardiovascular disease parameters in psoriasis patients over 52 weeks. J Invest Dermatol. 2019;139(5):1054–62. 30508547 10.1016/j.jid.2018.10.042

[22] Ahlehoff O, Hansen PR, Gislason GH, Frydland M, Bryld LE, Elming H, et al. Myocardial function and effects of biologic therapy in patients with severe psoriasis: a prospective echocardiographic study. J Eur Acad Dermatol Venereol. 2016;30(5):819–23. doi: 10.1111/jdv.13152 25845841

[23] Herédi E, Végh J, Pogácsás L, Gáspár K, Varga J, Kincse G, et al. Subclinical cardiovascular disease and it’s improvement after long-term TNF-α inhibitor therapy in severe psoriatic patients. J Eur Acad Dermatol Venereol. 2016;30(9):1531–6. doi: 10.1111/jdv.13649 27393182

[24] Wu JJ, Poon K-YT, Channual JC, Shen AY-J. Association between tumor necrosis factor inhibitor therapy and myocardial infarction risk in patients with psoriasis. Arch Dermatol. 2012;148(11):1244–50. doi: 10.1001/archdermatol.2012.2502 22911151

[25] Wu JJ, Guérin A, Sundaram M, Dea K, Cloutier M, Mulani P. Cardiovascular event risk assessment in psoriasis patients treated with tumor necrosis factor-α inhibitors versus methotrexate. J Am Acad Dermatol. 2017;76(1):81–90. doi: 10.1016/j.jaad.2016.07.042 27894789

[26] Ryan C, Leonardi CL, Krueger JG, Kimball AB, Strober BE, Gordon KB, et al. Association between biologic therapies for chronic plaque psoriasis and cardiovascular events: a meta-analysis of randomized controlled trials. JAMA. 2011;306(8):864–71. doi: 10.1001/jama.2011.1211 21862748

[27] Tzellos T, Kyrgidis A, Zouboulis CC. Re-evaluation of the risk for major adverse cardiovascular events in patients treated with anti-IL-12/23 biological agents for chronic plaque psoriasis: a meta-analysis of randomized controlled trials. J Eur Acad Dermatol Venereol. 2013;27(5):622–7. doi: 10.1111/j.1468-3083.2012.04500.x 22404103

[28] Rungapiromnan W, Yiu ZZN, Warren RB, Griffiths CEM, Ashcroft DM. Impact of biologic therapies on risk of major adverse cardiovascular events in patients with psoriasis: systematic review and meta-analysis of randomized controlled trials. Br J Dermatol. 2017;176(4):890–901. doi: 10.1111/bjd.14964 27518205 PMC5412670

[29] Wang W, Wang C-Y, Wang S-I, Wei JC-C. Long-term cardiovascular outcomes in COVID-19 survivors among non-vaccinated population: a retrospective cohort study from the TriNetX US collaborative networks. EClinicalMedicine. 2022;53:101619. doi: 10.1016/j.eclinm.2022.101619 35971425 PMC9366236

[30] Singla S, Putman M, Liew J, Gordon K. Association between biological immunotherapy for psoriasis and time to incident inflammatory arthritis: a retrospective cohort study. Lancet Rheumatol. 2023;5(4):e200–7. doi: 10.1016/S2665-9913(23)00034-6 38251522

[31] Ogdie A, Alehashemi S, Love TJ, Jiang Y, Haynes K, Hennessy S, et al. Validity of psoriatic arthritis and capture of disease modifying antirheumatic drugs in the health improvement network. Pharmacoepidemiol Drug Saf. 2014;23(9):918–22. doi: 10.1002/pds.3677 25044030 PMC4149813

[32] Mital R, Bayne J, Rodriguez F, Ovbiagele B, Bhatt DL, Albert MA. Race and ethnicity considerations in patients with coronary artery disease and stroke: JACC focus seminar 3/9. J Am Coll Cardiol. 2021;78(24):2483–92. doi: 10.1016/j.jacc.2021.05.051 34886970

[33] Armstrong AW, Puig L, Joshi A, Skup M, Williams D, Li J, et al. Comparison of biologics and oral treatments for plaque psoriasis: a meta-analysis. JAMA Dermatol. 2020;156(3):258–69. doi: 10.1001/jamadermatol.2019.4029 32022825 PMC7042876

[34] Manja V, AlBashir S, Guyatt G. Criteria for use of composite end points for competing risks—a systematic survey of the literature with recommendations. J Clin Epidemiol. 2017;82:4–11. doi: 10.1016/j.jclinepi.2016.12.001 27965044

[35] Kaushik SB, Lebwohl MG. Psoriasis: which therapy for which patient: psoriasis comorbidities and preferred systemic agents. J Am Acad Dermatol. 2019;80(1):27–40. doi: 10.1016/j.jaad.2018.06.057 30017705

[36] Moride Y, Abenhaim L. Evidence of the depletion of susceptibles effect in non-experimental pharmacoepidemiologic research. J Clin Epidemiol. 1994;47(7):731–7. doi: 10.1016/0895-4356(94)90170-8 7722586

[37] Neimann AL, Shin DB, Wang X, Margolis DJ, Troxel AB, Gelfand JM. Prevalence of cardiovascular risk factors in patients with psoriasis. J Am Acad Dermatol. 2006;55(5):829–35. doi: 10.1016/j.jaad.2006.08.040 17052489

[38] Armstrong A, Harskamp C, Dhillon J, Armstrong E. Psoriasis and smoking: a systematic review and meta-analysis. Br J Dermatol. 2014;170(2):304–14. 24117435 10.1111/bjd.12670

[39] Kolliker Frers RA, Cosentino V, Tau J, Kerzberg EM, Urdapilleta A, Chiocconi M, et al. Immune-mediated inflammation promotes subclinical atherosclerosis in recent-onset psoriatic arthritis patients without conventional cardiovascular risk factors. Front Immunol. 2018;9:139. doi: 10.3389/fimmu.2018.00139 29535705 PMC5834432

[40] Martinez-Lopez A, Blasco-Morente G, Perez-Lopez I, Tercedor-Sanchez J, Arias-Santiago S. Studying the effect of systemic and biological drugs on intima-media thickness in patients suffering from moderate and severe psoriasis. J Eur Acad Dermatol Venereol. 2018;32(9):1492–8. doi: 10.1111/jdv.14841 29405437

[41] Hjuler KF, Bøttcher M, Vestergaard C, Bøtker HE, Iversen L, Kragballe K. Association between changes in coronary artery disease progression and treatment with biologic agents for severe psoriasis. JAMA Dermatol. 2016;152(10):1114–21. doi: 10.1001/jamadermatol.2016.1984 27385305

[42] Taleb S, Romain M, Ramkhelawon B, Uyttenhove C, Pasterkamp G, Herbin O, et al. Loss of SOCS3 expression in T cells reveals a regulatory role for interleukin-17 in atherosclerosis. J Exp Med. 2009;206(10):2067–77. doi: 10.1084/jem.20090545 19737863 PMC2757872

[43] Danzaki K, Matsui Y, Ikesue M, Ohta D, Ito K, Kanayama M. Interleukin-17A deficiency accelerates unstable atherosclerotic plaque formation in apolipoprotein E-deficient mice. Arterioscler Thromb Vasc Biol. 2012;32(2):273–80. 22116098 10.1161/ATVBAHA.111.229997

[44] Elnabawi YA, Dey AK, Goyal A, Groenendyk JW, Chung JH, Belur AD, et al. Coronary artery plaque characteristics and treatment with biologic therapy in severe psoriasis: results from a prospective observational study. Cardiovasc Res. 2019;115(4):721–8. doi: 10.1093/cvr/cvz009 30721933 PMC6432047

[45] Makavos G, Ikonomidis I, Andreadou I, Varoudi M, Kapniari I, Loukeri E, et al. Effects of Interleukin 17A inhibition on myocardial deformation and vascular function in psoriasis. Can J Cardiol. 2020;36(1):100-111. 31606265 10.1016/j.cjca.2019.06.021

[46] Engelbertsen D, Depuydt M, Verwilligen R, Rattik S, Levinsohn E, Edsfeldt A. IL-23R deficiency does not impact atherosclerotic plaque development in mice. J Am Heart Assoc. 2018;7(8):e008257. 29618473 10.1161/JAHA.117.008257 PMC6015431

[47] Fatkhullina AR, Peshkova IO, Dzutsev A, Aghayev T, McCulloch JA, Thovarai V, et al. An Interleukin-23-Interleukin-22 axis regulates intestinal microbial homeostasis to protect from diet-induced atherosclerosis. Immunity. 2018;49(5):943-957.e9. doi: 10.1016/j.immuni.2018.09.011 30389414 PMC6257980

[48] Sbidian E, Chaimani A, Guelimi R, Garcia-Doval I, Hua C, Hughes C, et al. Systemic pharmacological treatments for chronic plaque psoriasis: a network meta-analysis. Cochrane Database Syst Rev. 2023;7(7):CD011535. doi: 10.1002/14651858.CD011535.pub6 37436070 PMC10337265

[49] Poizeau F, Nowak E, Kerbrat S, Le Nautout B, Droitcourt C, Drici M-D, et al. Association between early severe cardiovascular events and the initiation of treatment with the Anti-Interleukin 12/23p40 antibody Ustekinumab. JAMA Dermatol. 2020;156(11):1208–15. doi: 10.1001/jamadermatol.2020.2977 32902568 PMC7489429

[50] Reddy M, Torres G, McCormick T, Marano C, Cooper K, Yeilding N, et al. Positive treatment effects of ustekinumab in psoriasis: analysis of lesional and systemic parameters. J Dermatol. 2010;37(5):413–25. doi: 10.1111/j.1346-8138.2010.00802.x 20536646

[51] Ikonomidis I, Papadavid E, Makavos G, Andreadou I, Varoudi M, Gravanis K, et al. Lowering Interleukin-12 activity improves myocardial and vascular function compared with tumor necrosis factor-a antagonism or cyclosporine in psoriasis. Circ Cardiovasc Imaging. 2017;10(9):e006283. doi: 10.1161/CIRCIMAGING.117.006283 28899951

[52] Eldirany SA, Ho M, Bunick CG. Structural basis for how biologic medicines bind their targets in psoriasis therapy. Yale J Biol Med. 2020;93(1):19–27. 32226331 PMC7087057

[53] de Brito M, Yiu ZZN. Cardiovascular safety of biologics targeting Interleukin (IL)-12 and/or IL-23: what does the evidence say? Am J Clin Dermatol. 2021;22(5):587–601. doi: 10.1007/s40257-021-00612-9 34292509

[54] Woods RH. Potential cerebrovascular accident signal for risankizumab: a disproportionality analysis of the FDA Adverse Event Reporting System (FAERS). Br J Clin Pharmacol. 2023;89(8):2386–95. doi: 10.1111/bcp.15581 36321844

[55] Egeberg A, Thyssen JP. Increased reporting of cerebrovascular accidents with use of risankizumab observed in the Food and Drug Administration Adverse Events Reporting System (FAERS). Br J Dermatol. 2023;188(6):793–4. doi: 10.1093/bjd/ljad039 36797979

