# Supplementary material for: Cardiovascular disease risk in patients with psoriasis receiving biologics targeting TNF-α, IL-12/23, IL-17, and IL-23: A population-based retrospective cohort study
Source: PLoS Med. 2025 Apr 17;22(4):e1004591. doi: 10.1371/journal.pmed.1004591 (PMC12052210; doi:10.1371/journal.pmed.1004591)
Supplement: S1 Text — (PDF) [file pmed.1004591.s001.pdf]

S1 Text. Data Acquisition.

TriNetX stores data on physical or virtual appliances located within an institution's data center, forming a federated network that enables query broadcasting and result aggregation. All data undergo quality assessment, including data cleaning, to ensure compliance with TriNetX standards, adherence to clinical terminologies, and rejection of non-conforming records.

De-identified in accordance with HIPAA standards, the data include demographics (HL7 Version 3), diagnoses (ICD-10-CM), procedures (ICD-9-CM, ICD-10-PCS, SNOMED, CPT), medications (RxNorm, VA National Formulary, ATC), and laboratory measurements (LOINC). Data from participating healthcare organizations (HCOs) are updated every 1, 2, or 4 weeks. However, as TriNetX aggregates data from open systems, medical encounters outside participating HCOs are not captured, potentially leading to incomplete longitudinal records for patients who receive care across multiple healthcare systems.

The network primarily includes data from the USA, with contributions from other regions, including India, Australia, Malaysia, Taiwan, Spain, the UK, and Bulgaria. To comply with legal and ethical guidelines, the identities of HCOs and their data sources remain confidential. TriNetX categorizes HCOs into specialty networks based on regional coverage and data characteristics:

- US Network: Exclusively contains data from the USA.
- EMEA (Europe, Middle East, and Africa) Network: Includes data from Bulgaria, Germany, Italy, Lithuania, Malaysia, Poland, Spain, and the UK.

- APAC (Asia-Pacific) Network: Includes data from Australia, India, Malaysia, Singapore, and Taiwan.
- Global Network: The largest subset, incorporating data from all participating HCOs worldwide.
