# Supplementary material for: Cardiovascular disease risk in patients with psoriasis receiving biologics targeting TNF-α, IL-12/23, IL-17, and IL-23: A population-based retrospective cohort study
Source: PLoS Med. 2025 Apr 17;22(4):e1004591. doi: 10.1371/journal.pmed.1004591 (PMC12052210; doi:10.1371/journal.pmed.1004591)
Supplement: S2 Text — (PDF) [file pmed.1004591.s002.pdf]

S2 Text. Propensity Score Matching.

TriNetX performs cohort matching within its fixed platform. After defining the index date and covariates, the system conducts 1:1 propensity score matching without replacement. Propensity scores, ranging from 0 to 1, are calculated using logistic regression via scikit-learn, representing the probability of a patient belonging to the cohort of interest.

Matching follows a greedy nearest neighbor approach with a caliper of 0.1 pooled standard deviations, preventing matches between patients with widely differing scores. For each patient in the BIO-cohort, the system identifies the closest unmatched patient in the Non-BIO-cohort. Once matched, both patients are marked, and the process continues until all possible matches are assigned.
