# Supplementary material for: Cardiovascular disease risk in patients with psoriasis receiving biologics targeting TNF-α, IL-12/23, IL-17, and IL-23: A population-based retrospective cohort study
Source: PLoS Med. 2025 Apr 17;22(4):e1004591. doi: 10.1371/journal.pmed.1004591 (PMC12052210; doi:10.1371/journal.pmed.1004591)
Supplement: S3 Text — (PDF) [file pmed.1004591.s003.pdf]

S3 Text. Handling of Missing Data.

The TriNetX platform provides real-world data extracted from electronic medical records (EMRs) as entered by HCOs. Since data are collected during routine clinical care, completeness cannot be guaranteed. TriNetX does not impute or estimate missing clinical values, except in four specific cases:

- Patient Encounters

If an observation is recorded with an encounter ID that does not match any ID on the HCO's encounter list, TriNetX derives an encounter to accommodate these observations. If multiple observations share the same unmatched encounter ID, a single derived encounter is created, with the earliest observation date assigned as the start date and the latest as the end date. However, the encounter type is not inferred.

- Missing Start or End Dates for Encounters

If an HCO-provided encounter is missing a start date, TriNetX assigns the earliest start date from all associated observations. If an end date is missing, the latest date from related observations is assigned.

- Glomerular Filtration Rate (GFR) Estimation

Estimated GFR values are derived when serum, plasma, or blood creatinine levels are recorded, provided the patient's sex and age are known. For pediatric patients, a height measurement within one year of creatinine testing is required.

- Oncology Diagnoses

TriNetX maps oncology diagnoses from ICD-O codes to ICD-10-CM using the National Cancer Institute’s Surveillance, Epidemiology, and End Results (SEER) program guidelines. Diagnoses derived through this mapping are flagged accordingly.

Beyond these exceptions, missing data remain unaltered in the dataset, reflecting the original records as documented by the HCOs.
