# Supplementary material for: Cardiovascular disease risk in patients with psoriasis receiving biologics targeting TNF-α, IL-12/23, IL-17, and IL-23: A population-based retrospective cohort study
Source: PLoS Med. 2025 Apr 17;22(4):e1004591. doi: 10.1371/journal.pmed.1004591 (PMC12052210; doi:10.1371/journal.pmed.1004591)
Supplement: S1 Table — (PDF) [file pmed.1004591.s004.pdf]

S1 Table. Codes for all diagnoses, laboratory tests, and medications in our analysis

Specific data	Standardized code
Diagnoses	ICD-10-CM code
Psoriasis	L40
Rheumatoid arthritis	M05, M06
Ankylosing spondylitis	M45
Juvenile idiopathic arthritis	M08
Inflammatory bowel disease	K50, K51
Hidradenitis suppurativa	L73.2
Uveitis	H20, H30, H44.11
Stroke	I60-I69
Transient ischemic attacks	G45
Atrial fibrillation and flutter	I48
Tachycardia	R00.0, I47
Bradycardia	R00.1, I49.5, I49.8
Ventricular arrhythmias	I49
Pericarditis	I30-I32
Myocarditis	I40, I41, I51.4
Acute coronary disease	I24
Myocardial infarction	I21, I22
Ischemic cardiomyopathy	I25.5
Angina	I20
Heart failure	I50
Non-ischemic cardiomyopathy	I42
Pulmonary embolism	I26
Superficial or deep vein thrombosis	I80.0-I80.3, I80.8, I80.9, I81, I82
Peripheral arterial occlusive disease	I73.81, I73,89, I73.9, I74.01, I74.09, , I74.11, I74.2-I74.5, I74.8, I74.9, I79.1, I79.8
Cardiac arrest	I46
Cardiogenic shock	R57.0
Essential hypertension	I10
Chronic obstructive pulmonary disease	J44
Liver diseases	K70-K77
Chronic kidney disease	N18
Type 2 diabetes mellitus	E11
Hyperlipidemia	E78.5
Depression	F32
Sleep disorders	G47

Overweight or obesity	E66
Tobacco use	Z72.0
Nicotine dependence	F17
Socioeconomic challenges	Z55-Z65
Measurements	LOINC code
Body mass index	39156-5
Triglyceride in blood	12951-0, 2571-8, 3043-7
LDL-C in blood	13457-7, 18261-8, 18262-6, 2089-1, 49132-4
HDL-C in blood	18263-4, 2085-9, 49130-8,
CRP in blood	1988-5, 30522-7, 71426-1
ESR in blood	30341-2, 4537-7, 4538-5
Medications	RxNorm, VA or ATC code
Etanercept	RxNorm:214555
Adalimumab	RxNorm:327361
Infliximab	RxNorm:191831
Golimumab	RxNorm:819300
Certolizumabb pegol	RxNorm:709271
Ustekinumab	RxNorm:847083
Secukinumab	RxNorm:1599788
Brodalumab	RxNorm:1872251
Ixekizumab	RxNorm:1745099
Guselkumab	RxNorm:1928588
Risankizumab	RxNorm:2166040
Tildrakizumab	RxNorm:2053436
Methotrexate	RxNorm:6851
Cyclosporine	RxNorm:3008
Acitretin	RxNorm:16818
Apremilast	RxNorm:1492727
Thrombolytics	VA:BL115
Antiplatelets	VA:BL117
Anticoagulants	VA:BL110
Antiarrhythmics	VA:CV300, ATC:C01B
Antianginals	VA:CV250
Vasodilators used in cardiac diseases	ATC:C01D
β-blockers	ATC:C07
Cardiac-selective calcium channel blockers	ATC:C08D
ACEIs or ARBs	ATC:C09
Diuretics	ATC:C03

Sympathomimetics	VA:AU100
Digitalis glycoside	VA:CV050
Cilostazol	RXNORM:21107
Procedures	ICD-9-CM, ICD-10-PCS, SNOMED or CPT code
Cardiac catheterization	ICD-9-CM:36.06, 36.07<br>SNOMED: 36969009, 41976001, 429639007, 609154002, 609153008, 726011000<br>CPT:92928, 92929, 92933, 92934, 1021165, 1021166<br>HCPCS:C9602
Coronary artery bypass graft	ICD10PCS:0210093, 0210493, 02100A3, 02100Z3, 02100J3, 02100K3, 02104A3, 02104J3, 02104K3, 02104Z3<br>SNOMED:232717009, 232719007, 232720001, 232721002, 232722009, 232723004<br>CPT:1006200, 33511-33513, 33516, 33518, 33521
Pacemaker implantation	CPT:1006075

ICD-10-CM, International Classification of Diseases, Tenth Revision, Clinical Modification; LOINC, Logical Observation Identifiers Names and Codes, national health insurance; LDL-C, low density lipoprotein cholesterol; HDL-C, high density lipoprotein cholesterol; CRP, C reactive protein; ESR, erythrocyte sedimentation rate; VA, Veterans Affairs National Formulary; ATC, Anatomical Therapeutic Chemical Classification System; ACEIs, angiotensin-converting enzyme inhibitors; ARBs, angiotensin II receptor blockers; ICD-9-CM, International Classification of Diseases, Ninth Revision, Clinical Modification; ICD-10-PCS, International Classification of Diseases, Tenth Revision, Procedure Coding System; SNOMED, Systematized Nomenclature of Medicine; CPT, Current Procedural Terminology.
