# Supplementary material for: Cardiovascular disease risk in patients with psoriasis receiving biologics targeting TNF-α, IL-12/23, IL-17, and IL-23: A population-based retrospective cohort study
Source: PLoS Med. 2025 Apr 17;22(4):e1004591. doi: 10.1371/journal.pmed.1004591 (PMC12052210; doi:10.1371/journal.pmed.1004591)
Supplement: S2 Table — (PDF) [file pmed.1004591.s005.pdf]

S2 Table. Demographic characteristics of study subjects before PSM matching

	BIO-cohort (N = 30,215)	Non-BIO-cohort (N = 13,962)	Std. diff.*
Age, years, mean±SD			
Current age	52.5±15.0	58.2±16.5	0.363
Age at index	46.2±14.6	51.6±16.1	0.352
Female, N (%)	14,651 (48.7%)	6,601 (48.8%)	0.001
Race, N (%)†			
White	21,192 (70.5%)	7,005 (51.8%)	0.391
Black or African American	1,160 (3.9%)	452 (3.3%)	0.028
Asian	1,609 (5.4%)	2,017 (14.9%)	0.321
Unavailable	4,672 (15.5%)	3,619 (26.8%)	0.277
Comorbidity, N (%)			
Essential hypertension	4,745 (15.8%)	2,372 (17.5%)	0.047
Chronic obstructive pulmonary disease	341 (1.1%)	267 (2.0%)	0.068
Liver diseases	1,402 (4.7%)	497 (3.7%)	0.049
Chronic kidney disease	406 (1.4%)	240 (1.8%)	0.034
Type 2 diabetes mellitus	2,139 (7.1%)	1,021 (7.5%)	0.017
Hyperlipidemia	2,765 (9.2%)	1,451 (10.7%)	0.051
Depression	2,290 (7.6%)	849 (6.3%)	0.053
Sleep disorders	2,448 (8.1%)	981 (7.3%)	0.033
Overweight or obesity	3,206 (10.7%)	1,151 (8.5%)	0.073
Smoking, N (%)‡			
Tobacco use	447 (1.5%)	148 (1.1%)	0.035
Nicotine dependence	1,656 (5.5%)	635 (4.7%)	0.037
Socioeconomic challenges, N (%)‡	290 (1.0%)	105 (0.8%)	0.020
Laboratory measurements§			
Body mass index (kg/m2), mean±SD	31.3±7.7	30.4±7.4	0.116
≥27 kg/m2, N (%)	9,851 (32.8%)	3,554 (26.3%)	0.143
TG in blood (mg/dL), mean±SD	146.7±106.6	140.4±98.8	0.061
≥500 mg/dL, N (%)	207 (0.7%)	58 (0.4%)	0.035
LDL-C in blood (mg/dL), mean±SD	109.4±35.8	108.6±35.4	0.021

≥190 mg/dL, N (%)	277 (0.9%)	104 (0.8%)	0.017
HDL-C in blood (mg/dL), mean±SD	49.6±18.5	51.0±19.9	0.072
<40 mg/dL	2,551 (8.5%)	986 (7.3%)	0.044
CRP in blood (mg/L), mean±SD	10.7±22.9	10.4±24.8	0.012
>3 mg/L, N (%)	4,017 (13.4%)	1,320 (9.8%)	0.113
ESR in blood (mm/h), mean±SD	17.6±17.9	19.3±19.3	0.094
>25 mm/h, N (%)	1,798 (6.0%)	599 (4.4%)	0.070

PSM, propensity score matching; BIO-cohort, biologic cohort; Non-BIO-cohort, non-biologic cohort; Std. diff., standardized difference; SD, standard deviation; N, number; TG, triglyceride; LDL-C, low density lipoprotein cholesterol; HDL-C, high density lipoprotein cholesterol; CRP, C reactive protein; ESR, erythrocyte sedimentation rate.

*Cohorts were considered well-matched when standardized differences were below 0.1.

†Races not fully represented include the sparsely numbered American Indian, Alaska Native, Native Hawaiian, and other Pacific Islander.

‡Persons with tobacco use, nicotine dependence, and potential health hazards related to socioeconomic and psychosocial circumstances were identified with ICD-10-CM Z72.0, F17, and Z55-Z65, respectively.

§Laboratory test results were based on the most recent measurements prior to the index event.
