# Supplementary material for: Cardiovascular disease risk in patients with psoriasis receiving biologics targeting TNF-α, IL-12/23, IL-17, and IL-23: A population-based retrospective cohort study
Source: PLoS Med. 2025 Apr 17;22(4):e1004591. doi: 10.1371/journal.pmed.1004591 (PMC12052210; doi:10.1371/journal.pmed.1004591)
Supplement: S3 Table — (PDF) [file pmed.1004591.s006.pdf]

S3 Table. Outcome events among patients in the BIO-cohort and Non-BIO-cohort

	BIO-cohort (N = 12,732)	Non-BIO-cohort (N =12,732)	P-value
Any cardiovascular diseases	950 (7.5%)	1,281 (10.1%)	<0.001
Cerebrovascular diseases	225 (1.8%)	313 (2.5%)	<0.001
Arrhythmias	543 (4.3%)	726 (5.7%)	<0.001
Inflammatory heart diseases	31 (0.2%)	47 (0.4%)	0.070
Ischemic heart diseases	134 (1.1%)	199 (1.6%)	<0.001
Heart failure	165 (1.3%)	221 (1.7%)	0.004
Non-ischemic cardiomyopathy	59 (0.5%)	77 (0.6%)	0.122
Thrombotic disorders	102 (0.8%)	154 (1.2%)	0.001
Peripheral arterial occlusive disease	84 (0.7%)	144 (1.1%)	<0.001
Cardiac arrest or cardiogenic shock	23 (0.2%)	23 (0.2%)	1.000
Major adverse cardiac events	429 (3.4%)	525 (4.1%)	0.002
Use of medication for cardiovascular diseases			
Thrombolytics agents	61 (0.5%)	71 (0.5%)	0.383
Platelet aggregation inhibitors	760 (6.0%)	849 (6.7%)	0.022
Anticoagulants	1,760 (13.8%)	1,782 (14.0%)	0.690
Antiarrhythmic drugs	71 (0.6%)	93 (0.7%)	0.085
Antianginals	191 (1.5%)	248 (1.9%)	0.006
Vasodilators for cardiac diseases	190 (1.5%)	248 (1.9%)	0.005
Beta-blockers	933 (7.3%)	1,022 (8.0%)	0.036
Cardiac selective CCBs	88 (0.7%)	142 (1.1%)	<0.001
ACEIs or ARBs	1,831 (14.4%)	1,677 (13.2%)	0.005
Diuretics	1,279 (10.0%)	1,297 (10.2%)	0.708
Sympathomimetic drugs	1,502 (11.8%)	1,361 (10.7%)	0.005
Digitalis glycosides	12 (0.1%)	17 (0.1%)	0.353
Cilostazol	10 (0.1%)*	18 (0.1%)	0.130
Performing cardiovascular procedures†	33 (0.3%)	39 (0.3%)	0.479

BIO-cohort, biologic cohort; Non-BIO-cohort, non-biologic cohort; N, number; CCBs, calcium channel blockers; ACEIs, angiotensin-converting enzyme inhibitors; ARBs, angiotensin II receptor blockers.

*To maintain anonymity, TriNetX reports a value of 10 when the number of observations is fewer than 10.

†Cardiovascular procedures included performing cardiac catheterization, coronary artery bypass graft, and pacemaker implantation.
