# Supplementary material for: Cardiovascular disease risk in patients with psoriasis receiving biologics targeting TNF-α, IL-12/23, IL-17, and IL-23: A population-based retrospective cohort study
Source: PLoS Med. 2025 Apr 17;22(4):e1004591. doi: 10.1371/journal.pmed.1004591 (PMC12052210; doi:10.1371/journal.pmed.1004591)
Supplement: S4 Table — (PDF) [file pmed.1004591.s007.pdf]

S4 Table. Risk of specific category of cardiovascular diseases in subgroups prescribed single class biologics versus those on conventional systemic anti-psoriatic drugs

	Event/N.		
Study outcomes	BIO-cohort	Non-BIO-cohort	Hazard ratio (95% CI)
Use of only anti-TNF-α			
Cerebrovascular diseases	191/8,776	187/8,776	0.960 (0.785, 1.175)
Arrhythmias	500/8,776	539/8,776	0.867 (0.768, 0.979)
Inflammatory heart diseases	23/8,776	34/8,776	0.635 (0.374, 1.079)
Ischemic heart diseases	134/8,776	130/8,776	0.968 (0.761, 1.232)
Heart failure	122/8,776	137/8,776	0.834 (0.653, 1.064)
Non-ischemic cardiomyopathy	39/8,776	54/8,776	0.677 (0.449, 1.022)
Thrombotic disorders	98/8,776	117/8,776	0.788 (0.603, 1.031)
Peripheral arterial occlusive disease	98/8,776	92/8,776	1.002 (0.754, 1.332)
Cardiac arrest or cardiogenic shock	25/8,776	18/8,776	1.301 (0.710, 2.384)
Major adverse cardiac events	351/8,776	343/8,776	0.960 (0.827, 1.114)
Use of only anti-IL-12/23			
Cerebrovascular diseases	41/1,766	50/1,766	0.712 (0.471, 1.077)
Arrhythmias	105/1,766	92/1,766	0.994 (0.751, 1.315)
Inflammatory heart diseases	12/1,766	10/1,766	1.172 (0.494, 2.782)
Ischemic heart diseases	25/1,766	25/1,766	0.881 (0.506, 1.534)
Heart failure	29/1,766	31/1,766	0.825 (0.497, 1.369)
Non-ischemic cardiomyopathy	10/1,766*	15/1,766	0.519 (0.227, 1.186)
Thrombotic disorders	14/1,766	20/1,766	0.609 (0.308, 1.206)
Peripheral arterial occlusive disease	11/1,766	15/1,766	0.649 (0.298, 1.413)
Cardiac arrest or cardiogenic shock	10/1,766*	10/1,766*	0.580 (0.164, 2.056)
Major adverse cardiac events	76/1,766	77/1,766	0.864 (0.629, 1.186)
Use of only anti-IL-17			
Cerebrovascular diseases	41/2,950	43/2,950/2,950	0.835 (0.544, 1.280)
Arrhythmias	118/2,950	134	0.763 (0.596, 0.977)
Inflammatory heart diseases	10/2,950*	12/2,950	0.442 (0.166, 1.179)
Ischemic heart diseases	28/2,950	29/2,950	0.844 (0.502, 1.418)
Heart failure	26/2,950	40/2,950	0.559 (0.341, 0.916)
Non-ischemic cardiomyopathy	22/2,950	11/2,950	1.763 (0.855, 3.636)
Thrombotic disorders	24/2,950	29/2,950	0.719 (0.418, 1.235)
Peripheral arterial occlusive disease	19/2,950	25/2,950	0.664 (0.366, 1.207)
Cardiac arrest or cardiogenic shock	10/2,950*	10/2,950*	0.445 (0.081, 2.430)
Major adverse cardiac events	79/2,950	75/2,950	0.922 (0.672, 1.265)
Use of only anti-IL-23			

Cerebrovascular diseases	24/2,362	27/2,362	0.773 (0.446, 1.340)
Arrhythmias	101/2,362	116/2,362	0.750 (0.574, 0.979)
Inflammatory heart diseases	10/2,362*	11/2,362	0.080 (0.010, 0.619)
Ischemic heart diseases	20/2,362	20/2,362	0.877 (0.472, 1.630)
Heart failure	21/2,362	27/2,362	0.670 (0.379, 1.185)
Non-ischemic cardiomyopathy	10/2,362*	14/2,362	0.373 (0.143, 0.972)
Thrombotic disorders	26/2,362	28/2,362	0.812 (0.476, 1.385)
Peripheral arterial occlusive disease	10/2,362*	12/2,362	0.311 (0.139, 0.699)
Cardiac arrest or cardiogenic shock	10/2,362*	0/2,362	NA
Major adverse cardiac events	55/2,362	58/2,362	0.822 (0.569, 1.190)

N, number; BIO-cohort, biologic cohort; Non-BIO-cohort, non-biologic cohort; CI, confidence interval; NA, not available.

*To maintain anonymity, TriNetX reports a value of 10 when the number of observations is fewer than 10.
