# Supplementary material for: Cardiovascular disease risk in patients with psoriasis receiving biologics targeting TNF-α, IL-12/23, IL-17, and IL-23: A population-based retrospective cohort study
Source: PLoS Med. 2025 Apr 17;22(4):e1004591. doi: 10.1371/journal.pmed.1004591 (PMC12052210; doi:10.1371/journal.pmed.1004591)
Supplement: S5 Table — (PDF) [file pmed.1004591.s008.pdf]

S5 Table. Cardiovascular disease risk in patients with psoriasis receiving a single biologic versus oral anti-psoriatic drugs after 1:1 propensity score matching

	Hazard ratio (95% CI)											
	ETA (N = 2,358)	ADA (N = 6,012)	INF (N = 539)	GOL (N = 217)	CER (N = 188)	UST (N = 1,776)	SEC (N = 1,464)	IXE (N = 1,094)	BRO (N = 56)	GUS (N = 714)	TIL (N = 96)	RIS (N = 1,540)
Any CVDs	1.01 (0.84, 1.21)	0.93 (0.82, 1.05)	**1.55 (1.12, 2.13)**	0.70 (0.36, 1.35)	1.80 (0.79, 4.07)	0.92 (0.74, 1.13)	**0.76 (0.59, 0.99)**	1.09 (0.79, 1.51)	0.58 (0.10, 3.46)	1.23 (0.85, 1.79)	0.67 (0.28, 1.59)	**0.75 (0.56, 0.99)**
CeVDs	1.05 (0.72, 1.55)	0.96 (0.74, 1.24)	1.27 (0.67, 2.42)	1.50 (0.44, 5.14)	0.96 (0.19, 4.78)	0.71 (0.47, 1.08)	0.48 (0.27, 0.85)	0.98 (0.51, 1.88)	0.99 (0.06, 15.79)	1.19 (0.52, 2.71)	0.64 (0.11, 3.82)	**0.47 (0.23, 0.94)**
Arrhythmias	1.01 (0.80, 1.28)	0.97 (0.83, 1.13)	1.57 (1.04, 2.37)	0.31 (0.10, 0.97)	3.70 (1.03, 13.25)	0.99 (0.75, 1.32)	0.89 (0.63, 1.25)	1.14 (0.75, 1.75)	0.28 (0.03, 2.73)	1.18 (0.74, 1.86)	0.38 (0.10, 1.47)	0.76 (0.53-1.10)
InHDs	1.09 (0.39, 3.00)	0.67 (0.37, 1.22)	2.07 (0.19, 22.80)	NA*	NA*	1.17 (0.49, 2.78)	1.25 (0.21, 7.46)	0.88 (0.18, 4.35)	NA*	0.23 (0.03, 2.02)	NA*	NA*
IsHDs	0.88 (0.56, 1.39)	0.93 (0.68, 1.28)	1.02 (0.46, 2.28)	1.28 (0.21, 7.64)	5.08 (0.59, 43.48)	0.88 (0.51, 1.53)	1.09 (0.50, 2.40)	1.48 (0.58, 3.77)	NA*	1.06 (0.36, 3.17)	0.92 (0.06, 14.65)	NA*
Heart failure	0.76 (0.48, 1.21)	0.83 (0.60, 1.15)	1.64 (0.80, 3.38)	0.29 (0.06, 1.42)	NA*	0.82 (0.50, 1.37)	0.59 (0.27, 1.27)	0.76 (0.29, 1.98)	NA*	1.50 (0.55, 4.14)	NA*	0.66 (0.30, 1.44)
NICM	0.73 (0.32, 1.67)	0.57 (0.33, 1.00)	1.82 (0.53, 6.22)	NA*	NA*	0.52 (0.23, 1.19)	1.96 (0.60, 6.36)	3.50 (0.74, 16.48)	NA*	0.45 (0.04, 4.92)	0.89 (0.06, 14.24)	0.54 (0.25, 1.19)
Thrombotic disorders	1.12 (0.68, 1.86)	0.87 (0.61, 1.24)	2.45 (0.94, 6.38)	1.83 (0.17, 20.16)	NA*	0.61 (0.31, 1.21)	1.41 (0.62, 3.23)	0.66 (0.23, 1.91)	NA*	1.29 (0.49, 3.39)	NA*	0.58 (0.16, 2.05)
PAOD	1.12 (0.64, 1.96)	0.96 (0.66, 1.39)	0.95 (0.43, 2.08)	0.57 (0.10, 3.41)	0.95 (0.06, 15.20)	0.65 (0.30, 1.41)	**0.30 (0.13, 0.72)**	1.43 (0.52, 3.94)	NA*	0.74 (0.20, 2.75)	0.91 (0.13, 6.50)	1.08 (0.53, 2.20)
CA or CS	1.34 (0.42, 4.21)	0.74 (0.36, 1.55)	3.09 (0.32, 29.70)	NA*	NA*	0.58 (0.16, 2.06)	0.43 (0.04, 4.80)	NA	NA*	NA	NA*	0.47 (0.16, 1.39)
MACE	1.00 (0.75, 1.33)	1.06 (0.88, 1.29)	1.56 (0.95, 2.57)	0.87 (0.34, 2.19)	3.05 (0.62, 15.13)	0.86 (0.63, 1.19)	0.94 (0.60, 1.47)	1.11 (0.64, 1.92)	0.47 (0.04, 5.21)	1.32 (0.70, 2.50)	0.22 (0.03, 1.97)	1.69 (0.15, 18.61)

ETA, etanercept; ADA, adalimumab; INF, infliximab; GOL, golimumab; CER, certolizumab pegol; UST, Ustekinumab; SEC, secukinumab; IXE, ixekizumab; BRO, brodalumab; GUS, guselkumab; TIL, tildrakizumab; RIS Risankizumab; N, number; CVDs, cardiovascular diseases; CeVDs, cerebrovascular diseases; CI, confidence interval; InHDs, inflammatory heart diseases; IsHDs, ischemic heart disease; NICM, non-ischemic cardiomyopathy; PAOD, peripheral arterial occlusive disease; CA, cardiac arrest; CS, cardiogenic shock; MACE, major adverse cardiac events; NA, not available .

*The hazard ratio cannot be calculated because there are no events observed in either the biologic users or oral anti-psoriatic drug users.
