# Supplementary material for: Cardiovascular disease risk in patients with psoriasis receiving biologics targeting TNF-α, IL-12/23, IL-17, and IL-23: A population-based retrospective cohort study
Source: PLoS Med. 2025 Apr 17;22(4):e1004591. doi: 10.1371/journal.pmed.1004591 (PMC12052210; doi:10.1371/journal.pmed.1004591)
Supplement: S1 Fig — (PDF) [file pmed.1004591.s009.pdf]

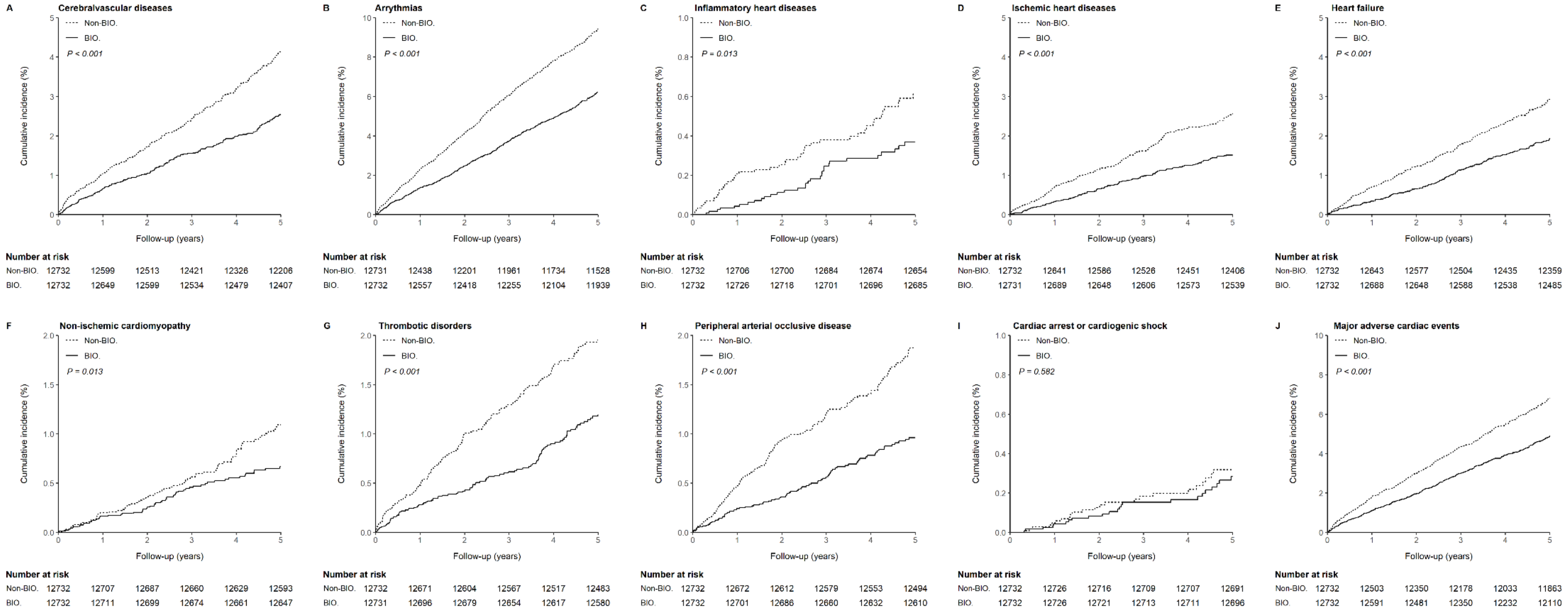

S1 Fig. Cumulative incidence of (A) cerebrovascular diseases, (B) arrhythmias, (C) inflammatory heart diseases, (D) ischemic heart diseases, (E) heart failure, (F) thrombotic disorders, (G) peripheral arterial occlusive disease, (H) cardiac arrest or cardiogenic shock, and (I) major adverse cardiac events in the BIOI-cohort versus the Non-BIO-cohort. The differences between the two study cohorts were determined by log-rank test. Abbreviation: BIO., biologic cohort; Non-BIO., non-biologic cohort.
