# Supplementary material for: Cardiovascular disease risk in patients with psoriasis receiving biologics targeting TNF-α, IL-12/23, IL-17, and IL-23: A population-based retrospective cohort study
Source: PLoS Med. 2025 Apr 17;22(4):e1004591. doi: 10.1371/journal.pmed.1004591 (PMC12052210; doi:10.1371/journal.pmed.1004591)
Supplement: S2 Fig — (PDF) [file pmed.1004591.s010.pdf]

Study outcomes	Event / N. BIO.	Event / N. Non-BIO.	Hazard ratio (95% CI)
Use of medication for cardiovascular diseases			
Thrombolytics	61/12,732	71/12,732	0.746 (0.530, 1.050)
Platelet aggregation inhibitors	760/12,732	849/12,732	0.769 (0.698, 0.848)
Anticoagulants	1,760/12,732	1,782/12,732	0.848 (0.794, 0.906)
Antiarrythmics	71/12,732	93/12,732	0.661 (0.485, 0.900)
Antianginals	191/12,732	248/12,732	0.663 (0.549, 0.801)
Vasodilators for cardiac diseases	190/12,732	248/12,732	0.659 (0.546, 0.796)
Beta-blockers	933/12,732	1,022/12,732	0.795 (0.727, 0.869)
Cardiac selective CCBs	88/12,732	142/12,732	0.545 (0.418, 0.711)
ACEIs or ARBs	1,831/12,732	1,677/12,732	0.965 (0.903, 1.031)
Diuretics	1,279/12,732	1,297/12,732	0.865 (0.801, 0.935)
Sympathomimetic drugs	1,502/12,732	1,361/12,732	0.948 (0.881, 1.020)
Digitalis glycosides	12/12,732	17/12,732	0.601 (0.287, 1.259)
Cilostazol	10/12,732	18/12,732	0.240 (0.089, 0.646)
Performing cardiovascular procedures	33/12,732	39/12,732	0.719 (0.452, 1.142)

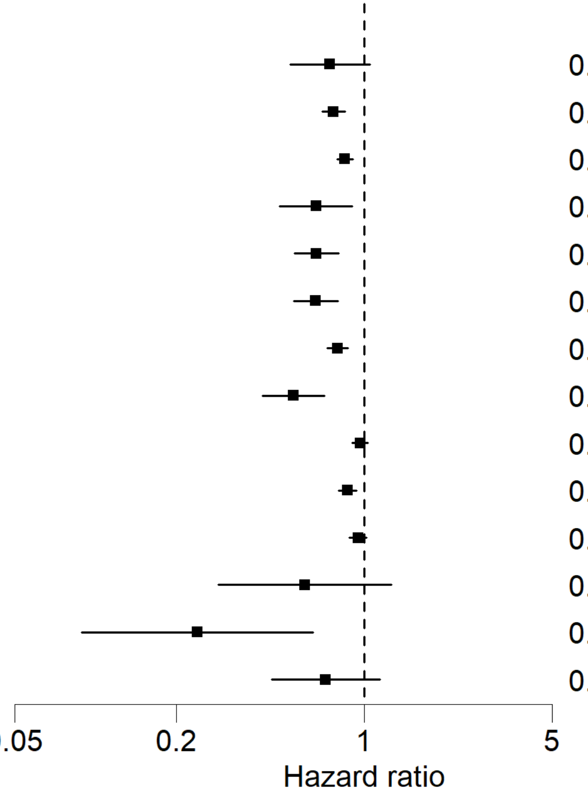

S2 Fig. Forest plot depicting hazard ratios for the utilization of cardiovascular disease-associated treatments. Cardiovascular procedures included performing cardiac catheterization, coronary artery bypass graft, and pacemaker implantation. Abbreviation: N, number; BIO., biologic cohort; Non-BIO., non-biologic cohort; CI, confidence interval; CCBs, calcium channel blockers; ACEIs, angiotensin-converting enzyme inhibitors; ARBs, angiotensin II receptor blockers.
