# Supplementary material for: Cardiovascular disease risk in patients with psoriasis receiving biologics targeting TNF-α, IL-12/23, IL-17, and IL-23: A population-based retrospective cohort study
Source: PLoS Med. 2025 Apr 17;22(4):e1004591. doi: 10.1371/journal.pmed.1004591 (PMC12052210; doi:10.1371/journal.pmed.1004591)
Supplement: S3 Fig — (PDF) [file pmed.1004591.s011.pdf]

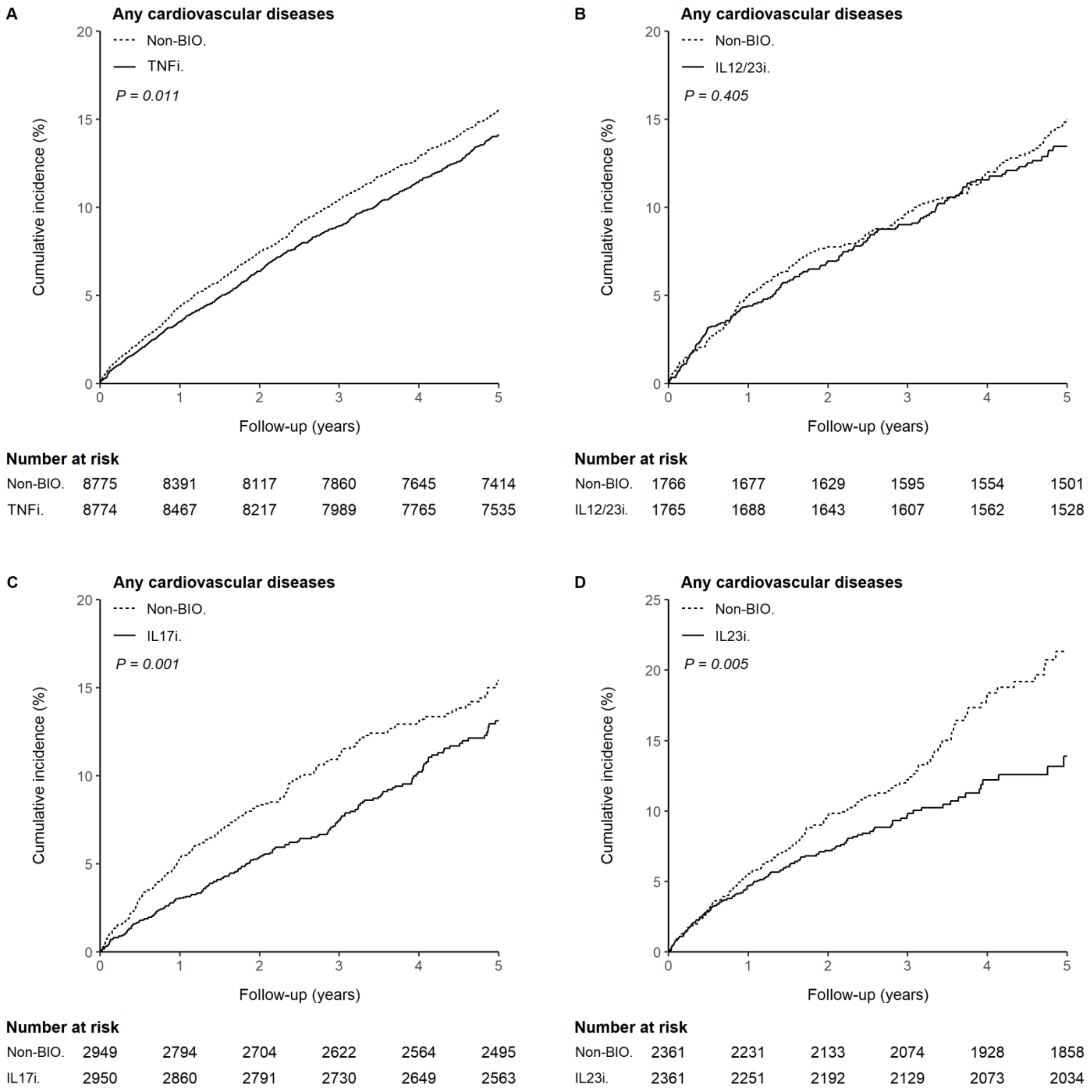

S3 Fig. Cumulative incidence of any cardiovascular diseases in the users of (A) anti- tumor necrosis factor-α, (B) anti-interleukin-12/23, (C) anti-interleukin-17, and (D) anti-interleukin-23 versus the Non-BIO-cohort. The differences between the two study cohorts were determined by log-rank test. Abbreviation: Non-BIO., non-biologic cohort; TNFi, tumor necrosis factor-α inhibitor users; IL12/23i, interleukin-12/23 inhibitor users; IL17i, interleukin-17 inhibitor users; IL23i, interleukin-23 inhibitor users.
