# Supplementary material for: Cardiovascular disease risk in patients with psoriasis receiving biologics targeting TNF-α, IL-12/23, IL-17, and IL-23: A population-based retrospective cohort study
Source: PLoS Med. 2025 Apr 17;22(4):e1004591. doi: 10.1371/journal.pmed.1004591 (PMC12052210; doi:10.1371/journal.pmed.1004591)
Supplement: S4 Fig — (PDF) [file pmed.1004591.s012.pdf]

Sensitivity analyses	Event / N. BIO.	Event / N. Non-BIO.	Hazard ratio (95% CI)
Model 1	855/12,732	1,126/12,732	0.628 (0.575, 0.686)
Model 2	1,038/12,732	1,445/12,732	0.600 (0.554, 0.650)
Model 3	767/10,353	1,009/10,353	0.654 (0.595, 0.718)
Model 4	897/11,163	1,130/11,163	0.667 (0.611, 0.729)
Model 5	708/9,084	909/9,084	0.627 (0.568, 0.692)
Model 6	950/12,732	1,281/12,732	0.621 (0.571, 0.676)
Model 7	996/12,714	1,280/12,714	0.651 (0.600, 0.708)
Model 8	3,103/16,195	3,269/16,195	0.830 (0.790, 0.871)

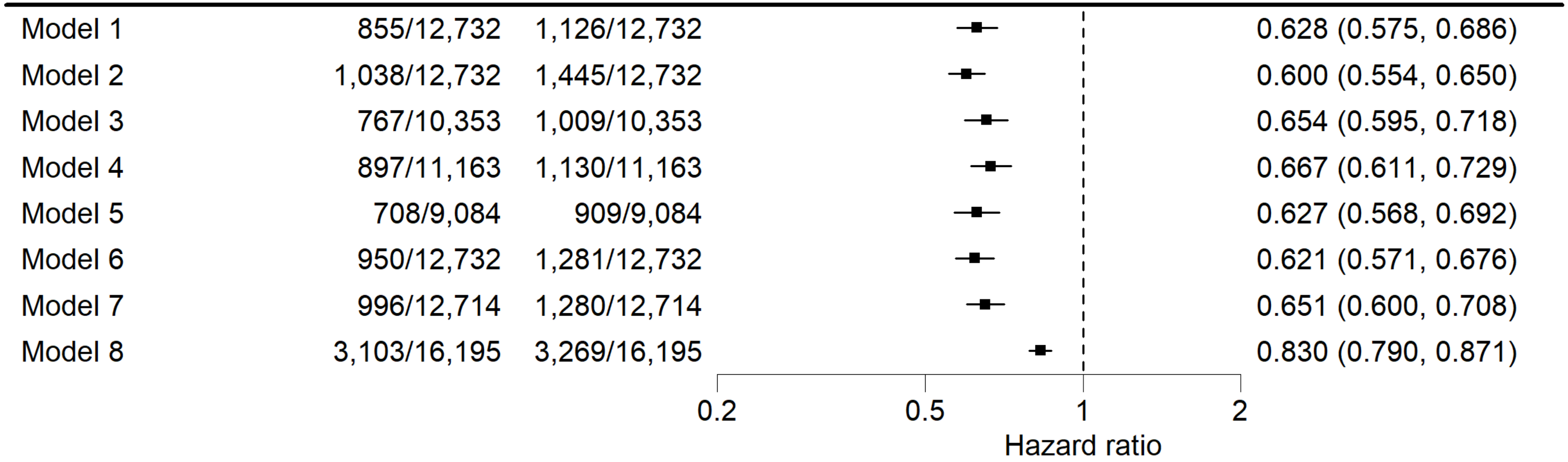

S4

S4 Fig. Forest plot depicting hazard ratios for the development of any cardiovascular diseases in eight sensitivity analyses. Model 1: one-year of washout period. Model 2: accounting for competing mortality. Model 3: excluding BIO-cohort patients previously prescribed conventional systemic anti-psoriatic drugs. Model 4: excluding cyclosporine users from the Non-BIO-cohort. Model 5: excluding apremilast users from the CONV-cohort. Model 6: adding a 6-month interval between two prescriptions for the definition of both cohorts. Model 7: including prior cardiovascular medications use as a matching condition. Model 8: including patients with prior cardiovascular diseases as additional matching criteria. Abbreviation: N, number; BIO., biologic cohort; Non-BIO., non-biologic cohort; CI, confidence interval.
