# Supplementary material for: Cardiovascular disease risk in patients with psoriasis receiving biologics targeting TNF-α, IL-12/23, IL-17, and IL-23: A population-based retrospective cohort study
Source: PLoS Med. 2025 Apr 17;22(4):e1004591. doi: 10.1371/journal.pmed.1004591 (PMC12052210; doi:10.1371/journal.pmed.1004591)
Supplement: S1 Checklist — (PDF) [file pmed.1004591.s013.pdf]

S1 Checklist. STROBE Statement.

	Item No	Recommendation	Section and paragraph
**Title and abstract**	1	(*a*) Indicate the study's design with a commonly used term in the title or the abstract<br>(*b*) Provide in the abstract an informative and balanced summary of what was done and what was found	Title and Abstract<br>Abstract, Methods and Finding
**Introduction**			
Background/rationale	2	Explain the scientific background and rationale for the investigation being reported	Paragraph 1-3
Objectives	3	State specific objectives, including any prespecified hypotheses	Introduction, paragraph 4
**Methods**			
Study design	4	Present key elements of study design early in the paper	Study Design and Population
Setting	5	Describe the setting, locations, and relevant dates, including periods of recruitment, exposure, follow-up, and data collection	Study Design and Population, Covariates, & Outcomes
Participants	6	(*a*) Give the eligibility criteria, and the sources and methods of selection of participants. Describe methods of follow-up<br>(*b*) For matched studies, give matching criteria and number of exposed and unexposed	Study Design and Population & Outcomes<br>Covariates
Variables	7	Clearly define all outcomes, exposures, predictors, potential confounders, and effect modifiers. Give diagnostic criteria, if applicable	Covariates, & Outcomes
Data sources/ measurement	8*	For each variable of interest, give sources of data and details of methods of assessment (measurement). Describe comparability of assessment methods if there is more than one group	Covariates
Bias	9	Describe any efforts to address potential sources of bias	Subgroup analyses & Sensitivity analyses
Study size	10	Explain how the study size was arrived at	Study Design and Population
Quantitative variables	11	Explain how quantitative variables were handled in the analyses. If applicable, describe which groupings were chosen and why	Covariates
Statistical methods	12	(*a*) Describe all statistical methods, including those used to control for confounding<br>(*b*) Describe any methods used to examine subgroups and interactions<br>(*c*) Explain how missing data were addressed<br>(*d*) If applicable, explain how loss to follow-up was addressed<br>(*e*) Describe any sensitivity analyses	Statistical analysis<br>Subgroup analyses<br>Statistical analysis<br>Outcomes<br>Sensitivity analyses
**Results**			
Participants	13*	(a) Report numbers of individuals at each stage of study—eg numbers potentially eligible, examined for eligibility, confirmed eligible, included in the study, completing follow-up, and analysed<br>(b) Give reasons for non-participation at each stage<br>(c) Consider use of a flow diagram	Demographic Characteristics<br>Demographic Characteristics<br>Demographic Characteristics
Descriptive data	14*	(a) Give characteristics of study participants (eg demographic, clinical, social) and information on exposures and potential confounders	Incidence and Risk of CVDs

			and Associated Therapy Utilization
		(b) Indicate number of participants with missing data for each variable of interest	Incidence and Risk of CVDs and Associated Therapy Utilization
		(c) Summarise follow-up time (eg, average and total amount)	Demographic Characteristics
Outcome data	15*	Report numbers of outcome events or summary measures over time	Incidence and Risk of CVDs and Associated Therapy Utilization
Main results	16	(*a*) Give unadjusted estimates and, if applicable, confounder-adjusted estimates and their precision (eg, 95% confidence interval). Make clear which confounders were adjusted for and why they were included	Incidence and Risk of CVDs and Associated Therapy Utilization
		(*b*) Report category boundaries when continuous variables were categorized	N/A
		(*c*) If relevant, consider translating estimates of relative risk into absolute risk for a meaningful time period	Incidence and Risk of CVDs and Associated Therapy Utilization
Other analyses	17	Report other analyses done—eg analyses of subgroups and interactions, and sensitivity analyses	Subgroup analyses Sensitivity analyses
**Discussion**			
Key results	18	Summarise key results with reference to study objectives	Paragraph 1
Limitations	19	Discuss limitations of the study, taking into account sources of potential bias or imprecision. Discuss both direction and magnitude of any potential bias	Paragraph 8
Interpretation	20	Give a cautious overall interpretation of results considering objectives, limitations, multiplicity of analyses, results from similar studies, and other relevant evidence	Paragraph 2-7
Generalisability	21	Discuss the generalisability (external validity) of the study results	Paragraph 6
**Other information**			
Funding	22	Give the source of funding and the role of the funders for the present study and, if applicable, for the original study on which the present article is based	Title page, p2

*Give information separately for exposed and unexposed groups.

Note: An Explanation and Elaboration article discusses each checklist item and gives methodological background and published examples of transparent reporting. The STROBE checklist is best used in conjunction with this article (freely available on the Web sites of PLoS Medicine at http://www.plosmedicine.org/, Annals of Internal Medicine at http://www.annals.org/, and Epidemiology at http://www.epidem.com/). Information on the STROBE Initiative is available at http://www.strobe-statement.org.
